# The Behavioral Presentation of Autistic Adults in a Forensic Interview

**DOI:** 10.1007/s10803-025-06805-z

**Published:** 2025-03-29

**Authors:** Katie Logos, Alliyza Lim, Neil Brewer, Robyn L. Young

**Affiliations:** 1 School of Social Sciences, University of Adelaide, Adelaide, Australia; 2 College of Education, Psychology & Social Work, Flinders University, Adelaide, Australia

**Keywords:** Autism spectrum disorder, Adult, Nonverbal communication, Verbal behavior, Diagnostic and Statistical Manual of Mental Disorders

## Abstract

Autism diagnostic criteria, and research primarily involving autistic children, highlight verbal and nonverbal behaviors likely to manifest during social interaction that may generate unfavorable impressions of interaction partners (e.g., poor credibility, incompetence). These behaviors are thought to bias evaluations of autistic individuals, particularly within high-stakes contexts (e.g., police or employment interviews). However, the prevalence of those behaviors in autistic adults is unclear. IQ-matched samples of autistic (*n* = 43) and non-autistic (*n* = 41) adults participated in a simulated chatroom, exposed to text-based conversations about illegal hacking. Participants were then interviewed about the chatroom in a one-on-one video-recorded online interview with the researcher. We measured the prevalence of 19 verbal and nonverbal behaviors, and memory report characteristics displayed by the interviewees, and investigated differences between the diagnostic groups. Diagnosis had a strong effect on overall behavioral displays but was only associated with minor differences in individual behaviors. Three significant effects indicated greater difficulty interpreting figurative language, longer speech hesitations, and greater verbal intonation for autistic than non-autistic adults. Inter-individual variability within groups and within-individual variability across behaviors highlighted that behaviors were neither ubiquitous nor consistently displayed in combination. There was also a suggestion of more noticeable differences in the behavior of male than female autistic adults. Although minor behavioral differences were detected based on diagnosis, they included behaviors that could lead to negative outcomes for autistic individuals during high-stakes interactions. Whether more pronounced behavioral differences are detected during face-to-face interactions warrants further research.

**Supplementary Information:**

The online version contains supplementary material available at 10.1007/s10803-025-06805-z.

## The Behavioral Presentation of Autistic Adults in a Forensic Interview

Recent research suggests that verbal and nonverbal behaviors exhibited by some autistic individuals may adversely affect how they are perceived during interpersonal interactions (Lim et al., 2022a; Norris et al., 2024; Sasson et al., 2017), though it is unclear precisely which behaviors, or combinations thereof, shape observers’ perceptions. Such behavioral effects have been observed in diminished credibility of crime suspects (Logos et al., 2021), unfavorable perceptions of interviewees during employment interviews (Flower et al., 2021; Norris et al., 2024), and poor impressions of likeability during ordinary interpersonal interactions (Sasson & Morrison, 2019). Promisingly, preliminary research has shown that education about autism-associated behaviors—based on diagnostic criteria—can moderate negative evaluations of autistic suspects interviewed during criminal investigations (e.g., Lim et al., 2022a; Logos et al., 2021). However, autism diagnostic criteria are primarily derived from how the condition presents in children. The manifestation of autism in adults—particularly during interpersonal interactions—is less well-documented and it is unclear how the behavioral presentations of autistic and non-autistic adults differ during social interactions. Yet, understanding the behavioral presentation of autistic adults during interactions in forensic contexts is a crucial prerequisite for (a) recognizing how those autism-associated behaviors may unfairly bias high-stakes judgments during such interactions, and (b) mitigating such biases through the development of appropriate educational tools. Consequently, we examined the presence of a range of verbal and nonverbal behaviors displayed by autistic and non-autistic adults when interviewed about their interactions in an online chatroom forum about cybercrime.

### Negative Interpersonal Judgments of Autistic Adults

Several studies have reported that people are more likely to deliver unjust negative interpersonal evaluations of autistic than non-autistic adults. Observers have judged autistic individuals (or those displaying autism-associated behavior) as less likeable, attractive, and approachable; more awkward and submissive; more deceptive and less credible; and less desirable interaction partners. These impressions were based on observation of images and short video clips (e.g., Lim et al., 2022b; Sasson & Morrison, 2019; Sasson et al., 2017), longer videos (Alkhaldi et al., 2019; Norris et al., 2024), and direct social interaction (Morrison et al., 2020). Studies have included high-stakes judgment contexts such as employment screening (Flower et al., 2021; Norris et al., 2024), and forensic interviews with witnesses, suspects, or defendants (Berryessa et al., 2015; Lim et al., 2022a; Logos et al., 2021; Maras et al., 2019).

Yet, the behaviors shaping such evaluations are unclear. Indeed, in one study (Lim et al., 2022a) in which behaviors considered likely to affect judgments of credibility and veracity—eye contact, repetitive body movement, emotional affect, verbal reciprocity, and literal interpretation of figurative language—were carefully operationalized and coded, no significant differences were detected in the behaviors of autistic (*n* = 30) and non-autistic (*n* = 29) witnesses during video testimony. Yet, autistic interviewees were judged as less credible and more deceptive. Moreover, those judgments were predicted by the overall clinical impressions of psychologists who were blind to the target’s diagnosis, suggesting some aspects of the targets’ behavioral presentation influenced observers’ evaluations.

Recently, Norris et al. (2024) found that observers rated autistic job candidates (*n* = 14) as having a more monotonous tone, being less composed and focused, and having reduced natural eye contact and gestures than their non-autistic counterparts (*n* = 18). However, they reported no group differences in perceptions of vocabulary, speech clearness and coherence, facial expression, body language, or tone or volume of voice. Norris et al. also found that while non-autistic interviewees were perceived as more confident and effective communicators when observers viewed a video record of the interview rather than just reading a transcript, this advantage did not accrue for autistic interviewees, suggesting that features of the behavioral presentation of autistic adults were influencing observers’ evaluations. In the current study we used systematic behavioral recording and coding of a range of behaviors to understand the presentation of autistic adults, thereby complementing studies that have focused on fewer behaviors or more subjective global impressions of behavior.

### Potential Behavioral Characteristics of Autistic Adults

A guide to behavioral characteristics that might be expected to distinguish autistic from non-autistic adults is provided by the Diagnostic and Statistical Manual of Mental Disorders – Fifth Edition, Text Revision (DSM-5-TR; American Psychiatric Association [APA], 2022) which considers autism as encompassing sustained “deficits" in social communication and interaction (Criteria A), and repetitive and stereotyped behaviors and interests (Criteria B). Here we focus on behaviors amenable to observational measurement in an interactive interview paradigm.

The social communication and interaction domain is marked by difficulties in social-emotional reciprocity, including a lack of reciprocal turn-taking during conversations, monologues on topics of interest or irrelevant and tangential speech, reduced use of speech, difficulty interpreting language and social cues, socially inappropriate speech, idiosyncratic language, and reduced expression of emotion (APA, 2022). These difficulties might be expected to be the most salient characteristics of autistic adults, with studies demonstrating increased monologues leading to one-sided approaches to conversation, literal interpretation of language, flat emotional affect or incongruous emotional expression, poor emotion recognition, and general pragmatic differences (Billstedt et al., 2007; Brewer et al., 2016; Kimura et al., 2020; Lim et al., 2022a). However, inter-individual variability is often marked (e.g., Georgopoulos et al., 2022; Lim et al., 2022a). Further, much of the research has involved small samples of autistic adults, is based on self-report surveys of behavior difficulties, or has examined the presence of behavior in non-naturalistic, non-interactive communicative contexts.

Nonverbal communicative behaviors including absent, irrelevant, reduced, excessive, and uncommon nonverbal behaviors, as well as integration with verbal behavior, have also attracted researchers’ attention (APA, 2022). Such behaviors include aspects of eye contact such as intense eye contact (Dalton et al., 2005; Pelphrey et al., 2002; Spezio et al., 2007) or avoiding eye contact (Klin et al., 2002; Neumann et al., 2006). Research into paralinguistic cues including body language and gestures suggests an overall reduced use of gestures by autistic individuals, but with great heterogeneity across individuals (McKern et al., 2023). However, the majority of this research examining nonverbal behaviors focused on children. In one study, self-reports indicated reduced body language by autistic adults may be misinterpreted by healthcare professionals who rely on such cues during social interactions, resulting in autistic people feeling that the seriousness of their medical condition was underestimated or misunderstood (Strömberg et al., 2022). Another observational study indicated more frequent use of interactive gestures and gestures that were more difficult to interpret for autistic than non-autistic adults (de Marchena et al., 2019). Although clinical observations and criteria highlight difficulties in appropriate nonverbal expression of emotion by autistic individuals, and studies have reported difficulties for autistic adolescents and adults in facial expression mimicry, unexpected and incongruent emotion expression and flatter emotional affect (Macdonald et al., 1989; McIntosh et al., 2006; Stel et al., 2008), limited empirical research has systematically examined spontaneous naturalistic displays of emotion by autistic adults during social communication. Differences in prosody evidenced in autistic adult samples include monotone intonation, irregular patterns of stress on words, reduced fluency, differences in control of pitch and intensity and vocal quality, decreased use of filled pauses through use of filler words (e.g., “um” and “uh”), and increased use of silent pauses (Irvine et al., 2016; Lake et al., 2011; Paul et al., 2005; Pirinen et al., 2023; Shriberg et al., 2001). However, these studies did not examine prosody within high-stakes contexts where participants are asked multiple specific questions regarding a personally experienced event—stressful contexts potentially influencing prosodic features (Buchanen et al., 2014).

With respect to DSM-5-TR Criteria B, the prevalence of repetitive and stereotyped behaviors—repetitive motor movements such as hand flapping or finger flicking, repetitive speech such as immediate echolalia, or stereotyped, idiosyncratic, or repetitive use of words or phrases—in autistic adults is unclear. In a meta-analytic review, stereotypical repetitive behaviors were variously reported in 21.9% to 97.5% of autism diagnoses (median = 51.8%; Melo et al., 2019), with increased prevalence associated with lower nonverbal IQ, greater “severity” of autism, and younger age. Yet there has been limited observational research with autistic adults examining the prevalence and trajectory of these behaviors. Some research suggests the prevalence of stereotyped repetitive behaviors decreases over time, though such behaviors may not completely disappear in adulthood (Ballaban-Gil et al., 1996; Barrett et al., 2018; Chowdhury et al., 2010). Repetitive patterns of speech during social interaction are also understudied in adults. For example, echolalia is a common behavior in autistic children (Ryan et al., 2022; Van Santen et al., 2013), but has not, to our knowledge, been systematically examined in comparisons of autistic and non-autistic adults. We do not discuss findings for other features of Criteria B—insistence on sameness, restricted and fixated interests, and sensory sensitivities—because they were either unlikely to emerge within our paradigm or presented difficulty for coding given likely inter-individual variability in the types of interests or sensitivities.

In sum, there is a dearth of research measuring the prevalence of verbal and nonverbal behaviors in autistic adults during interpersonal interactions that (a) are likely to shape how the individual is perceived and (b) were amenable to examination within our interview paradigm. Understanding the likely display of these behaviors in a forensic context is particularly important given many of the behaviors highlighted by diagnostic criteria also overlap with behavioral cues relied on by police, judges and jurors as indicators of poor credibility or deception (Strömwall & Granhag, 2003; Vrij & Turgeon, 2018). Research suggests it is unclear what, if any, behaviors can be reliably used in detecting lies (Luke, 2019). Yet, forensic decision makers still base their decisions on the display of these behaviors, which may lead to unjust outcomes for anyone displaying such behaviors—with autistic individuals perhaps at a greater disadvantage due to behavioral characteristics which are integral to the diagnosis.

In addition to behaviors suggested by DSM-5-TR criteria, differences in the episodic memory reports of autistic and non-autistic adults when interviewed have been noted, differences that might also shape perceivers’ impressions. Maras and colleagues (Maras & Bowler, 2011, 2012; Maras et al., 2020) reported that autistic adults provide less detailed and accurate information than non-autistic adults when questioned about an experienced or witnessed event. In contrast, Bagnall et al. (2023) failed to detect any difference in the number of accurate details provided by autistic and non-autistic adults but did note that autistic adult suspects were more likely to omit important details that could prove their innocence (see also, Young & Brewer, 2020).

The confidence individuals express in their memory reports is also known to be a powerful influence on perceivers’ judgments (e.g., Brewer & Burke, 2002). Although Maras et al. (2020) did not detect autistic-nonautistic differences in memory report confidence, autistic individuals have displayed lower confidence in their performance across various tasks (Carpenter & Williams, 2023; Georgopoulos et al., 2022). Consequently, we also examined characteristics of individuals’ memory reports, including associated confidence judgments, provided in our interview paradigm.

### The Study

We examined behavioral features displayed by autistic adults within an interaction simulating a potentially high-stakes situation. Autistic and non-autistic adults were interviewed regarding their involvement in a simulated chatroom where discussion of illegal hacking behavior took place. The study was conceived and conducted during COVID-19 lockdown periods and, consequently, used an online video-recorded interview format. We used rigorous behavioral coding to measure whether, during interpersonal interactions, autistic adults were characterized by a range of (a) verbal and nonverbal communicative behaviors and (b) repetitive and stereotyped behaviors that are aligned with diagnostic criteria. We sought to answer the following research questions: Are the behaviors reflected in autism diagnostic criteria likely to be displayed by autistic adults during social interaction that may occur in a forensic setting, and are these behaviors more prevalent than for non-autistic adults? Our aim was to advance understanding of the likely behavioral characteristics of autistic adults that may result in their being evaluated negatively, and unjustly, when involved in interpersonal interactions such as interviews. Further, we examined whether any behavioral differences between autistic and non-autistic individuals endured after controlling for symptoms of depression, anxiety, and stress, conditions that often co-occur with a diagnosis of autism (Hollocks et al., 2019; Nah et al., 2018) and may, individually or collectively, be associated with reduced emotion expression, increased repetitive movement, and memory reporting errors (Bylsma et al., 2008; Kizilbash et al., 2002; Lang et al., 2015).

Recent research notes gender differences in behaviors associated with autism. It has been suggested that diagnostic criteria align with the ‘typical’ autistic male presentation, and that autistic females may be less likely to exhibit some behaviors within a social context (Kreiser & White, 2014; Tsirgiotis et al., 2024). Some studies suggest this may be due to autistic females being more likely than autistic males to employ compensatory, masking, or ‘camouflaging’ strategies to adjust their socio-communicative behavior to meet ‘typical’ social expectations (e.g., Hull et al., 2020). Note, however, that the relevant findings are mixed and a number of methodological issues undermine these conclusions (Cook et al., 2021; Pearson & Rose, 2021). Therefore, we conducted an exploratory examination of whether the effect of autism diagnosis on the presence of each of the coded behaviors varied with participant self-reported gender.

## Methods

### Participants and Design

Participants in the cross-sectional study included 43 autistic adults and 41 non-autistic adults. Key demographic and ability characteristics are shown in Table 1, demonstrating that autistic and non-autistic participants did not differ on self-reported gender identity, age, ethnicity, measures of verbal intelligence as indicated by scores on the Wechsler Abbreviated Scale of Intelligence–Second Edition verbal comprehension index (WASI-II VCI; Minshew et al., 2005; Wechsler, 2011), or associative and episodic memory as measured by the Verbal Paired Associates task 15-item version (VPA-15; Uttl et al., 2002).

**Table 1:** Demographic and ability statistics for the autistic and non-autistic samples

Measure		Diagnosis	
		Autistic (n = 43)	Non-autistic (n = 41)
Gender			
	Female	46.5% (*n* = 20)	48.8% (*n* = 20)
	Male	53.5% (*n* = 23)	48.8% (*n* = 20)
	Non-binary	–	2.4% (*n* = 1)
	Chi-square statistic	χ^2^(2) = 1.16, *p* = .559, φ = .118, 95% CI [− .099, .324]	
Age			
	Range	18 to 69	18 to 62
	*M (SD)*	35.33 (13.57)	34.32 (10.51)
	*t*-test statistic	*t*(82) = 0.38, *p* = .705, *d* = 0.08, 95% CI [− 035, 0.51]	
Ethnicity			
	Caucasian	97.7% (*n* = 42)	100% (*n* = 41)
	Non-Caucasian	2.3% (*n* = 1)	0%
	Chi-square statistic	χ^2^(1) = 0.00, *p* = 1.00, φ = .107, 95% CI [− .110, .314]	
Verbal intelligence			
	Range	79 to 144	82 to 151
	*M (SD)*	113.84 (15.68)	112.24 (14.71)
	*t*-test statistic	*t*(82) = 0.48, *p* = .633, *d* = 0.11, 95% CI [− 032, 0.53]	
Verbal paired-associates (VPA)*			
	Range	0.5 to 15	1.5 to 14.5
	*M (SD)*	8.39 (4.38)	8.77 (4.21)
	*t*-test statistic	*t*(80) = 0.40, *p* = .692, *d* = 0.09, 95% CI [− 0.35, 0.52]	

* = Two participants did not complete the measure of VPA, for cell sizes of autistic (*n* = 41) and non-autistic (*n* = 41)

Participants self-selected to participate after reviewing the study details. Approximately half of the autistic (*n* = 23) and non-autistic participants (*n* = 18) were recruited via an advertisement sent out to databases of individuals who had indicated prior interest in research participation with the host university. To be included on the database, autistic participants were required to have an official diagnosis of autism by a qualified clinician. The remainder of the autistic (*n* = 20) and non-autistic (*n* = 23) participants were recruited via the online crowd-sourcing platform, Prolific. Participants complete an eligibility study to access the Prolific platform and they are also invited to complete a detailed checklist of information which researchers may use for screening potential participants. One pre-screening question focused on autism diagnosis. When the study was conducted around 2,200 of 120,000 potential participants were registered as autistic. All Prolific autistic participants reported receiving a formal diagnosis of autism from a registered diagnostician (i.e., speech pathologist, psychologist, pediatrician, or psychiatrist) or registered multidisciplinary team, with further diagnosis details gauged verbally during the Zoom session and confirmed by clinical members of the research team as consistent with assessments for autism (see Supplementary Materials S1). Further sample characteristics, including information on descriptive measures providing support for the integrity of the autism diagnostic classification, are summarized in the Results section.

Informed consent was obtained prior to participation. Information regarding the behavioral focus of the study, and the conduct of autistic-non-autistic comparisons, was withheld until the debriefing stage to prevent potential demand characteristics from biasing behavior—though it was possible that some comparative aspect of the research could be deduced by participants who were recruited on the basis of their diagnosis. Participants were informed that they could withdraw at any time, skip any questions or tasks without penalty, and were provided with contact details for support services and the opportunity to discuss their participation with the researcher during the debriefing stage. All these (and other) aspects of the study were approved by the Flinders University Social and Behavioral Ethics Committee, approval number 1850.

### Measures

#### Autism Classification

To provide support for the integrity of the autism diagnostic classification, other descriptive measures of autistic traits, adaptive behavior, and theory of mind were included. Autistic traits were measured using the Autism Quotient 10 (AQ-10; Allison et al., 2012), an abbreviated variant of the AQ-50 screening tool for autism (Baron-Cohen et al., 2001). Scores range from 0 to 10; higher scores indicate a greater presence of autistic traits. The Adaptive Behavior Assessment System 3rd Edition (ABAS-3; Harrison & Oakland, 2015) measured participants’ adaptive skill across three domains (practical, social, and conceptual) and provided a guide to functional difficulty level in these areas. Scores across domains are scaled and combined to produce a General Adaptive Composite (GAC) score ranging from 40 to 120; higher scores indicate greater adaptive skills. Theory of mind (ToM)—one’s ability to infer the mental states of others—was measured with the six-item social subscale of the Adult Theory of Mind (A-ToM) test, requiring participants to interpret the real meaning of various real-life social interactions they viewed within video scenarios; a comprehensive psychometric evaluation of the A-ToM is provided in Brewer et al. (2017). Scores range from 0 to 12; higher scores indicate stronger ToM.

#### Depression, Anxiety, and Stress

Given the greater likelihood of co-occurring depressive and anxiety disorders among autistic people, and potential links between these conditions and the presence of certain verbal and nonverbal behaviors, a measure of depression, anxiety and stress was used as a covariate when assessing relationships between diagnosis and behavior presentation. The Depression Anxiety and Stress Scale 21 (DASS-21; Lovibond & Lovibond, 1995; Park et al., 2020) required participants to rate their experienced emotional distress over the week prior to their participation by responding to statements capturing the domains of depression, anxiety and stress. Composite Total scores range from 0 to 126; higher scores indicate greater overall experienced depression, anxiety, and stress (Henry & Crawford, 2005).

### Procedure

Participants were told the study was examining how individuals discuss cybercrime. They participated individually in a simulated group chatroom discussion about cybercrime with other pseudo-participants, followed by a one-on-one filmed video-conference interview with a researcher about that discussion, conducted in the style of a police interview.

To ensure participants had sufficient knowledge of cybercrime to participate in the chatroom and answer interview questions, they (a) were provided with written information on hacking (i.e., what it is, how it works, and how to protect yourself from it), and (b) answered a quiz about that information administered via Qualtrics (with 100% accuracy required to continue; see Supplementary Materials S2). Participants then entered a text-only chatroom under instructions to discuss the hacking information provided with three other participants whom they understood to be legitimate participants. However, the other ‘participants’ were pre-written responses entered by the researcher, with one of the other ‘participants’ designated as the moderator who questioned participants in the chatroom. Suspicion regarding the legitimacy of other ‘participants’ was expected given the anonymous nature of the chatroom, with a follow-up question at the completion of the study indicating 25% of participants believed these were legitimate participants, 31% were unsure and 44% did not. Importantly, there was no difference in suspicion between autistic and non-autistic participants (see Supplementary Materials S3). The chatroom lasted approximately 10 min and involved discussion of each chatroom member’s experience of being hacked, including an admission of ‘White Hat’ hacking from the moderator (i.e., paid hacking undertaken to find security breaches for a company), as well as several sarcastic interactions between two of the pseudo-participants with one jokingly taunting the other to hack them (see Supplementary Materials S3 for chatroom script). The majority of the chatroom experience involved the real participant reading through the responses of the other pseudo-participants in real-time. At the end of the chatroom the moderator asked one question of the real participant regarding their experience of being hacked; following the participant’s response, the conversation ended.

Participants were then linked onto Zoom and interviewed by the researcher about their demographic characteristics (3 questions), hacking knowledge (3 questions based on the information they received as part of the study), and the chatroom interaction (14 questions). Participants were informed the interview would be conducted in the manner of a police interview during an investigation of suspicious online activity. They were not explicitly informed to modify their behavior for this context, but asked to answer honestly and in as much detail as they could remember—as would be the case during a real investigation. To ensure behavioral cues could be captured during the interview, participants were requested to be seated to allow for the top of their head to their knees to be visible within the frame. Interviews ranged from 6 to 17 min, with an average length of about 10 min. The latter section of the interview included questions regarding what the participant thought the intent was behind some of the other pseudo-participants’ statements in the chatroom, their own honesty in answering the moderator’s question, and how much they trusted the responses of the other pseudo-participants (see Supplementary Materials S4 for interview schedule). Interviews were video recorded, with the recordings later transcribed by a research assistant.

Following the interview, the researcher administered the WASI-II VCI and VPA-15. Participants were then directed back to Qualtrics to complete the DASS-21, AQ-10, ABAS-3 and A-ToM social subscale. Debriefing took place at the end of the study via Qualtrics, at which time participants were informed of the true purpose of the research, provided the opportunity to contact or Zoom with the researchers if they had any follow-up questions, and provided details for mental health resources should they feel any discomfort. Participants were also provided with the option to remove their recorded interview from use in further research (one of the participants selected this).

### Behavior and Memory Report Coding

Interview recordings and transcriptions were coded quantitatively for various behaviors—all of which are listed and operationalized in Table 2—that were selected on the basis of those outlined within Criteria A and B of the DSM-5-TR diagnostic criteria for autism. Only behaviors observable within the online interview context were coded. In line with Criteria A, seven aspects of verbal behavior relevant to social-emotional reciprocity and six aspects of nonverbal behavior were coded—noting there may be some overlap for behaviors between these categories. For Criteria B, the domain of repetitive and stereotyped patterns of behavior, four behaviors were coded. Additionally, to account for the likely variability in the appearance of some nonverbal behaviors, the intensity of four behaviors was coded as subtle, moderate, or intense based on the visible extent of the behavior (e.g., repetitive finger tapping (subtle), repetitive hand tapping (moderate) or repetitive arm raising (intense). Complete coding guidelines, including examples and non-examples of each behavior and the recording method (e.g., frequency recording, partial or full interval recording), are presented in Supplementary Materials S5.

**Table 2:** Behaviors, their operationalization, and reliability, coded in line with DSM-5-TR criteria

DSM-5-TR criteria	Behavior	Operationalization
Criteria A: social-emotional reciprocity		
1	Verbal tangents	Total duration (in seconds) that the articulation was either (1) completely irrelevant to the topic of conversation, or (2) relevant to the general topic of conversation but not necessary to answer the interviewer’s question. ICC = .790 [.559, .907]
2	Verbal hesitations	Mean duration (in seconds) of silent pauses (lasting more than 0.5 s) when it was their turn to speak. Coded with Praat
3	Articulation rate	Mean speed at which participant relayed their speech during the interview. Number of syllables uttered, divided by phonation time. Coded with Praat
4	Verbal fillers	Number of meaningless sound(s) used during speech to fill silence. ICC = .952 [.878, .982]
5	Literal interpretation	Degree to which interview responses suggested interpretation of the statement in an overly literal manner, indicating that the speaker’s intention when using sarcasm, innuendo and colloquialisms was not understood. Scores ranged from 0 to 6, with higher scores indicating more literal interpretation. ICC = .816 [.575, .927]
6	Inappropriate utterances	Number of verbalizations that were rude, unsuitable, or improper for the context of the interaction. ICC = .782 [.506, .912]
7	Idiosyncratic language	Number of sentences using standard words or phrases in an unusual way, such that the intended meaning was not readily apparent. ICC = N/A due to no occurrence of behavior
Criteria A: nonverbal communicative behaviors		
8 & 9	Gaze (maintenance and number of shifts)	Percentage of the interview that gaze was maintained directly into the camera (or where the interviewer’s face would be assumed to appear on the individual’s screen). ICC = .949 [.870, .981] Percentage of the interview when gaze was averted from the gaze maintenance area. ICC = .934 [.832, .975]
10	Representational gestures (presence and intensity)	Percentage of the interview that deliberate and relevant movements of the body were made when expressing ideas or as a replacement for speech. ICC = .777 [.498, .910] Percentage of the interview that intense relevant gestures were displayed. ICC = .744 [.436, .896]
11	Non- representational gestures (presence and intensity)	Coded as above for representational gestures not connected to the verbal statement. Presence ICC = .911 [.779, .966], intensity ICC = .954 [.882, .983]
12	Verbal intonation	Standard deviation associated with the mean vocal pitch, which indicated the level of vocal variation/affect used in verbalizations. Coded with Praat
13	Facial expression (presence and intensity)	Percentage of the interview when the face changed from the neutral baseline expression to a positive or negative expression. ICC = .890 [.730, .957] Percentage of the interview that intense facial expression was displayed. ICC = .890 [.731, .957]
Criteria B: repetitive and stereotyped behaviors		
14	Repetitive motor movement (presence and intensity)	Percentage of the interview that motor movement (not including eye movement) or use of objects was displayed that (1) was not required to meet the demands of the interview or did not appear to serve a functional purpose, and (2) was displayed more than once successively within the duration of the interview. ICC = .853 [.650, .942] Percentage of the interview that intense repetitive motor movement was displayed. ICC = .887 [.724, .956]
15 & 16	Verbal speech repeats (one word) or restarts (> one word)	Number of meaningless repetitions of words or phrases immediately after their initial occurrence. A single word repeated = a repeat, ICC = .808 [.557, .924]; a phrase repeated = a restart, ICC = .755 [.457, .901]
17	Echolalia (immediate)	Number of repetitions of words or phrases immediately after being spoken by the interviewer. ICC = N/A due to no occurrence of behavior

‘ICC’ is intraclass correlation coefficient [and 95% confidence intervals], single measures, consistent agreement

Characteristics of participants’ memory reports coded quantitatively from their interview responses (see Supplementary Materials S6) included the duration of their report (measured by the length of the interview in seconds), accurate and inaccurate reporting of information provided to participants during the study (i.e., as part of the hacking information or chatroom), information not provided to them during the study (i.e., based on existing knowledge), and expressions of uncertainty in any of the details reported.

### Reliability

The majority of behaviors and all memory characteristics were coded manually by one independent non-autistic coder (a Clinical Psychology PhD student) after extensive training and checking by two of the researchers, with interrater reliability examined using a second non-autistic coder (a study co-author) analyzing approximately 25% of interviews for each item coded. Prior to this, extensive coding instructions were developed and trialed across four training videos to establish coder agreement and sound reliability. For these fourteen behaviors (and four intensity ratings) and all memory characteristics, intraclass correlation coefficients ranged from 0.744 to 0.954, *p* < 0.001, across all items with consistent agreement between coders. The remaining three behaviors (hesitations, intonation, articulation rate) were coded using speech features extracted by automated audio software Praat (Boersma & Weenink, 2007) from the clipped audio recordings of interviewee responses (removing the interviewer’s questions), including pitch (Hz), number of syllables uttered, and speech pauses (see Supplementary Materials S5 for further detail on Praat coding procedures). Given interviews were conducted via video-conference, recording quality was dependent upon participants’ internet connections. As there were instances where video quality could not capture nonverbal behaviors, or audio quality could not adequately capture verbal behaviors or content, sample sizes for analyses varied across behaviors and memory reports—with a total loss of no more than six autistic (14%) and three non-autistic participants (7%) for any measure.

### Analytic Strategy

Note that our sample sizes are relatively small, perhaps reflecting prospective participants’ preparedness to consent under the condition that the videotaped interviews would be used as stimulus material in subsequent studies. Consequently, our focus in data interpretation was on effect size indices and their associated confidence intervals (cf. Gardner & Altman, 1986; Sullivan & Feinn, 2012), complemented by examination of Bayes factors to indicate support for the null hypothesis for any non-significant group contrasts.

First, a series of *t*-tests were used to examine characteristics of the sample, including whether descriptive measures provided further support for the integrity of the autism diagnosis, and whether autistic and non-autistic adults differed on DASS-21 Total scores. Next, a series of *t*-tests were used to compare memory report indices between groups to ensure that any detected differences in behavior between autistic and non-autistic adults were not simply a function of the length of time for which participants spoke or their ability to recall details.

Then a one-way MANOVA examined whether diagnosis had an effect on the presence of each of the coded behaviors (except for idiosyncratic language and immediate echolalia which were observed in three and zero participants, respectively). The multivariate analysis involved listwise deletion for all behaviors, reducing the sample size to 35 in each diagnosis group. Therefore, follow-up analyses involving separate one-way ANOVAs assessed the effect of autism diagnosis on each of the 19 behaviors, with inspection of effect sizes and Bayes factors to reduce the possibility of failing to detect potentially meaningful group differences. Further, a series of ANCOVAs were carried out to adjust for DASS-21 Total scores as a covariate.

Correlation coefficients were calculated between each behavior, separately for the autistic and non-autistic participants, to examine the relationships between behaviors and whether they differed based on diagnosis. Additionally, an exploratory 2 (diagnosis: autism, no autism) × 2 (gender: male, female) MANOVA was carried out to examine the effect of gender, with a series of separate follow-up one-way ANOVAs to examine the effect of diagnosis on behavior separately for male and female participants (note only one participant identified as non-binary, so they were not included in these analyses).

Across analyses, all non-normally distributed continuous variables were transformed using square root or log transformations where required (and as noted within analyses). Although inferential tests used those transformed variables, the descriptive statistics presented are the raw non-transformed values (see Supplementary Materials S7 for transformed descriptive statistics). When interpreting effect sizes and Bayes factors we followed the guidelines suggested by Fritz et al. (2012) and Lee and Wagenmakers (2014), respectively. All analyses were conducted using SPSS v.28 (IBM, 2022) and JASP (JASP Team, 2024).

## Results

### Sample Characteristics

Sample characteristics appear in Table 3. Autistic adults reported experiencing greater autistic traits on the AQ-10 and poorer adaptive skills on the ABAS-3 than non-autistic adults, demonstrating a strong effect of diagnosis. Although autistic adults did not perform significantly worse than non-autistic adults on the A-ToM social subscale, the effect size was consistent with the moderate (and significant) group differences reported in studies with much larger samples (e.g., Brewer et al., 2017, 2022). Similarly, the distributional characteristics aligned with those studies’ findings that very low ToM scores are largely confined to only a relatively small proportion of autistic adults. The effect of diagnosis across these three measures—in conjunction with the absence of a group difference in verbal IQ as measured by the WASI-II (see Table 1)—is consistent with the diagnostic classification and provided support for the integrity of the diagnosis. Additionally, consistent with previous research, the autistic sample reported greater combined depression, anxiety, and stress on the DASS-21 Total than the non-autistic sample*.*

**Table 3:** Sample characteristics

Measure		Diagnosis	
		Autistic (n = 43)	Non-autistic (n = 41)
AQ-10			
	Range	2 to 10	2 to 8
	*M (SD)*	6.84 (2.18)	2.78 (1.97)
	*t*-test statistic	*t*(82) = 8.93, *p* < .001, *d* = 1.95 [1.41, 2.45]	
ABAS (GAC)			
	Range	61 to 117	52 to 120
	*M (SD)*	88.05 (12.68)	102.56 (15.41)
	*t*-test statistic	*t*(82) = 4.73, *p* < .001, *d* = 1.03 [0.57, 1.48]	
A-ToM Social			
	Range	3 to 12	6 to 12
	*M (SD)*	9.21 (2.00)	10.00 (1.64)
	*t*-test statistic	*t*(72) = 1.85, *p* = .068, *d* = 0.43 [− 0.03, 0.89]	
DASS-21 (Total)			
	Range	0 to 62	0 to 53
	*M (SD)*	49.15 (33.34)	26.98 (21.28)
	*t*-test statistic	*t*(71.80) = 3.65, *p* < .001*, d* = 0.79 [0.34, 1.23]	

### Memory Report

Autistic and non-autistic adults did not differ significantly on any of the memory report indices, with effect sizes ranging from negligible to weak (see Table 4).

**Table 4:** Mean (SD) and t-test results comparing autistic and non-autistic adults on memory report characteristics

Memory report	Diagnosis		*t*-test results
	Autistic (n = 43)	Non-autistic (n = 41)	
Interview duration (seconds)^tL^	578.79 (142.18)	561.71 (164.83)	*t*(82) = 0.70, *p* = .486, *d* = 0.15 [− 0.28, 0.58]
Total details^tL^	66.60 (22.62)	69.67 (19.85)	*t*(80) = 0.98, *p* = .332, *d* = 0.22 [− 0.22, 0.65]
Total accurate details^tL^	61.21 (21.30)	64.56 (19.43)	*t*(80) = 1.06, *p* = .292, *d* = 0.24 [− 0.20, 0.67]
Accurate study detail	48.35 (14.42)	53.54 (13.80)	*t*(80) = 1.66, *p* = .101, *d* = 0.37 [− 0.07, 0.80]
Inaccurate study detail^tS^	4.72 (2.90)	4.72 (2.26)	*t*(80) = 0.06, *p* = .955, *d* = 0.10 [− 0.42, 0.45]
Accurate additional detail^tL^	12.86 (9.94)	11.03 (9.16)	*t*(80) = 1.15, *p* = .255, *d* = 0.25 [− 0.18, 0.69]
Uncertainty expressed^tL^	10.98 (6.94)	9.62 (7.52)	*t*(80) = 1.31, *p* = .195, *d* = 0.29 [− 0.15, 0.72]

^tL^transformed to normal distribution using log transformation

^t^^S^transformed to normal distribution using square root transformation

There were so few inaccurate additional details that this variable was not examined

The non-autistic sample size was 39 for all measures except for interview duration

### Behavior Patterns

The MANOVA results indicated a main effect of autism diagnosis, *F*(19, 50) = 2.18, *p* = .014, η^2^ = 0.453. For the ANOVA follow-up analyses, descriptive statistics are displayed in Table 5, and ANOVA results in Table 6, organized according to whether the direction of group differences was consistent or inconsistent with expectations based on diagnostic criteria. Behavioral differences are ordered within each of these categories according to their effect size. Table 6 also summarizes the Bayes factor outcomes and interpretations for each of the behaviors to demonstrate which of the non-significant outcomes with very small effect sizes reflected evidence in favor of the null hypothesis. We note that applying a Bonferroni correction to the alpha level to reduce the likelihood of a type I error (viz. *p* = .003) rendered the contrasts for all behaviors nonsignificant. Therefore, we present the uncorrected group contrasts so as not to mask any potential group differences due to the small sample size (cf. Nakagawa, 2004), with our focus primarily on the effect size indices, corresponding confidence intervals, and Bayes factors to reduce the likelihood of a type I error. Further, we note that the series of ANCOVAs adjusting for DASS-21 scores revealed negligible differences compared to the unadjusted results, so only the unadjusted results are reported (see Supplementary Materials S8 for a comparison of adjusted and unadjusted scores).

**Table 5:** Mean (SD) for individual behaviors between groups, categorized by patterns consistent and inconsistent with predictions based on diagnostic criteria

Behavior pattern	Diagnosis	
	Autistic	Non-autistic
Consistent with diagnostic criteria		
Verbal hesitations (mean seconds)^tL^	1.38 (0.36), *n* = 43	1.20 (0.17), *n* = 39
Literal interpretation (range 0–6)^tS^	0.79 (1.08), *n* = 43	0.31 (0.61), *n* = 39
Facial expression present %	51.59 (20.58), *n* = 39	59.01 (21.35), *n* = 40
Facial expression intensity %	26.62 (13.89), *n* = 39	29.93 (14.29), *n* = 40
Gaze shifts %	8.95 (3.62), *n* = 37	9.70 (2.87), *n* = 39
Gaze maintenance %	60.11 (18.78), *n* = 37	63.75 (16.13), *n* = 39
Verbal tangents (total seconds)* ^tL^	7.93 (22.21), *n* = 43	11.79 (39.85), *n* = 39
Verbal speech restarts (count of > one word)^tL^	1.07 (3.86), *n* = 43	0.44 (0.99), *n* = 39
Repetitive motor movement present %	67.25 (25.01), *n* = 41	63.23 (31.57), *n* = 38
Inappropriate utterances^tS^	0.09 (0.48), *n* = 43	0.05 (0.32), *n* = 39
Verbal fillers^tS^	39.37 (23.29), *n* = 43	42.23 (30.38), *n* = 39
Repetitive motor movement intensity %	31.16 (14.49), *n* = 41	31.03 (17.71), *n* = 38
Inconsistent with diagnostic criteria		
Verbal intonation (SD of pitch)^tL^	64.61 (25.57), *n* = 43	52.12 (22.76), *n* = 39
Representational gestures intensity %	12.62 (6.24), *n* = 43	10.75 (5.78), *n* = 41
Verbal repeats (count of one word)^tS^	0.74 (1.36), *n* = 43	1.44 (2.50), *n* = 39
Representational gestures present %	22.12 (9.24), *n* = 43	19.78 (8.97), *n* = 41
Non-representational gestures present %	28.09 (15.19), *n* = 43	29.57 (15.41), *n* = 41
Articulation rate (mean speed)	4.28 (0.33), *n* = 43	4.26 (0.28), *n* = 39
Non-representational gestures intensity %	17.06 (10.55), *n* = 43	17.74 (11.17), *n* = 41

^tL^ transformed using log transformation

^t^^S^ transformed using square root transformation

* The untransformed descriptive statistics for verbal tangents indicate longer overall tangent duration for the non-autistic group. However, analysis of the transformed values reversed this relationship, with longer verbal tangents for the autistic group (see Supplementary Materials S7 for transformed descriptive statistics)

**Table 6:** ANOVA and Bayesian outcomes for the effect of autism diagnosis on behaviors for behavior patterns consistent and inconsistent with predictions based on diagnostic criteria

Behavior pattern	ANOVA outcome	Interpretation*
Behavior pattern consistent with diagnostic criteria		
More verbal hesitations	*F* (1, 80) = 7.29, *p* = .008	Significant
	η^2^ = .084 [.006, .212]	Medium effect
	*BF*_*10*_ = 5.11	Moderate evidence for H_1_
More literal interpretation	*F* (1, 80) = 6.02, *p* = .016	Significant
	η^2^ = .070 [.002, .194]	Medium effect
	*BF*_*10*_ = 3.01	Moderate evidence for H_1_
Flatter facial expression	*F* (1, 77) = 2.48, *p* = .120	Non-significant
	η^2^ = .031 [.000, .137]	Weak effect
	*BF*_*10*_ = 0.68	Anecdotal evidence for H_0_
Flatter facial expression intensity	*F* (1, 77) = 1.09, *p* = .300	Non-significant
	η^2^ = .014 [.000, .103]	Weak effect
	*BF*_*10*_ = 0.38	Anecdotal evidence for H_0_
Fewer gaze shifts	*F* (1, 74) = 1.00, *p* = .320	Non-significant
	η^2^ = .013 [.000, .104]	Very weak effect
	*BF*_*10*_ = 0.37	Anecdotal evidence for H_0_
Less gaze maintenance	*F* (1, 74) = 0.82, *p* = .367	Non-significant
	η^2^ = .011 [.000, .098]	Very weak effect
	*BF*_*10*_ = 0.34	Anecdotal evidence for H_0_
Longer verbal tangents	*F* (1, 80) = 0.81, *p* = .372	Non-significant
	η^2^ = .010 [.000, .091]	Very weak effect
	*BF*_*10*_ = 0.33	Moderate evidence for H_0_
More verbal speech restarts	*F* (1, 80) = 0.66, *p* = .420	Non-significant
	η^2^ = .008 [.000, .085]	Negligible effect
	*BF*_*10*_ = 0.31	Moderate evidence for H_0_
More repetitive motor movement	*F* (1, 77) = 0.40, *p* = .530	Non-significant
	η^2^ = .005 [.000, .078]	Negligible effect
	*BF*_*10*_ = 0.28	Moderate evidence for H_0_
More inappropriate utterances	*F* (1, 80) = 0.21, *p* = .647	Non-significant
	η^2^ = .003 [.000, .065]	Negligible effect
	*BF*_*10*_ = 0.25	Moderate evidence for H_0_
Fewer verbal fillers	*F* (1, 80) = 0.09, *p* = .770	Non-significant
	η^2^ = .001 [.000, .053]	Negligible effect
	*BF*_*10*_ = 0.24	Moderate evidence for H_0_
More intense repetitive movement	*F* (1, 77) = 0.001, *p* = .972	Non-significant
	η^2^ = .000 [.000, .003]	Negligible effect
	*BF*_*10*_ = 0.23	Moderate evidence for H_0_
Behavior Pattern Inconsistent with Diagnostic Criteria		
More varied verbal intonation	*F* (1, 80) = 6.45, *p* = .013	Significant
	η^2^ = .075 [.003, .200]	Medium effect
	*BF*_*10*_ = 3.61	Moderate evidence for H_1_
More intense representational gestures	*F* (1, 82) = 2.02, *p* = .159	Non-significant
	η^2^ = .024 [.000, .120]	Very weak effect
	*BF*_*10*_ = 0.55	Anecdotal evidence for H_0_
Less repetitive speech	*F* (1, 80) = 1.43, *p* = .235	Non-significant
	η^2^ = .018 [.000, .109]	Very weak effect
	*BF*_*10*_ = 0.43	Anecdotal evidence for H_0_
More representational gestures	*F* (1, 82) = 1.39, *p* = .243	Non-significant
	η^2^ = .017 [.000, .105]	Very weak effect
	*BF*_*10*_ = 0.42	Anecdotal evidence for H_0_
Fewer non-representational gestures	*F* (1, 82) = 0.20, *p* = .658	Non-significant
	η^2^ = .002 [.000, .062]	Negligible effect
	*BF*_*10*_ = 0.25	Moderate evidence for H_0_
Slower articulation rate	*F* (1, 80) = 0.10, *p* = .754	Non-significant
	η^2^ = .001 [.000, .054]	Negligible effect
	*BF*_*10*_ = 0.25	Moderate evidence for H_0_
Less intense non- representational gestures	*F* (1, 82) = 0.08, *p* = .774	Non-significant
	η^2^ = .001 [.000, .051]	Negligible effect
	*BF*_*10*_ = 0.24	Moderate evidence for H_0_

*All effects are non-significant with Bonferroni corrections applied

Three group contrasts were statistically significant, supported by Bayes factors (BF_10_) providing moderate evidence for the alternative hypothesis (see Table 6). Two were consistent with predicted patterns for autistic adults: maintaining longer speech hesitations and interpreting speech more literally. The other significant contrast was contrary to expectations, with autistic adults producing more (rather than less) variation in their intonation.

For other contrasts that were not statistically significant, the effect sizes indicated effects ranging from weak to negligible. The Bayes factor (BF_10_) evidence from the non-significant contrasts ranged from anecdotal to moderate evidence in favor of the null hypothesis. Contrasts that only provided anecdotal evidence for the null hypothesis included behavioral trends either (a) consistent with predicted patterns, including flatter and less intense facial expression of emotion, increased gaze aversion and fewer gaze shifts for autistic adults, or (b) inconsistent with predicted patterns, including more representational and intense gestures integrated with their speech and less repetitive speech for autistic adults. Although these contrasts were non-significant and weak, the Bayes factors signal the possibility that significant group differences might be detected with larger sample sizes.

The Bayes factors for all other non-significant contrasts indicated moderate evidence for the null hypothesis. Thus, it seems unlikely that, even with much larger sample sizes, those remaining behaviors (i.e., verbal tangents, verbal restarts, repetitive motor movement, inappropriate utterances, verbal fillers, non-representational gestures not connected to speech and articulation rate) would differ significantly between autistic and non-autistic adult samples.

As indicated by the standard deviations in Table 5, there was considerable inter-individual variability within each group for most behaviors. Importantly, for the autistic group there was also considerable intra-individual variability across behaviors; the inter-correlation matrix for all behaviors for only the autistic participants appears in Table 7, with the corresponding matrix for non-autistic participants in Supplementary Materials S9. Several features of the correlation matrix merit comment. First, a number of strong correlations emerged between variables that would be expected to correlate: for example, between facial expression presence and intensity, repetitive movement presence and intensity, and presence and intensity of both representational and non-representational gestures. Second, four modest correlations were detected between verbal behaviors that might be expected to be related to some degree based on diagnostic criteria (e.g., between verbal tangents and both literal interpretation and fillers, and between verbal repeats and restarts). Third, and strikingly, behaviors that might be expected to be prominent among those associated with an autism diagnosis—such as literal interpretation, the presence and intensity of facial expressions, eye gaze behaviors and repetitive movements—were uncorrelated.

**Table 7:** Pearson r correlation [and 95% confidence intervals for r] between behaviors for autistic adults (n = 35)

	Verbal hesitations	Literal interpretation	Facial expression present	Facial expression intensity	Gaze shifts	Gaze maintenance	Verbal tangents	Verbal speech restarts	Repetitive motor movement	Inappropriate utterances	Verbal fillers	Repetitive motor intensity	Verbal intonation	Representational intensity	Verbal repeats	Representational present	Non-representational present	Articulation rate
Verbal hesitations^tL^																		
Literal interpretation^tS^	** − .137** [**− **.450, .205]																	
Facial expression present %	** − .272** [**− **.555, .067]	**.237** [**− **.105, .528]																
Facial expression intensity %	** − .323** [**− **.592, .012]	**.198** [**− **.145, .498]	.942*** [.887, .971]															
Gaze shifts %	** − .047** [**− **.374, .291]	**.256** [**− **.085, .543]	**.103** [**− **.239, .421]	**.140** [**− **.202, .452]														
Gaze maintenance %	**.057** [**− **.282, .383]	** − .212** [**− **.509, .130]	**.129** [**− **.213, .444]	**.113** [**− **.229, .430]	− .430* [**− **.668, − .113]													
Verbal tangents^tL^	** − .188** [**− **.491, .155]	.445** [.131, .678]	**.013** [**− **.321, .345]	** − .027** [**− **.357, .309]	**.205** [**− **.137, .504]	** − .321** [**− **.591, .014]												
Verbal speech restarts^tL^	** − .045** [**− **.372, .293]	**.274** [**− **.065, .556]	**.286** [**− **.052, .565]	**.256** [**− **.085, .543]	**.193** [**− **.150, .494]	** − .317** [**− **.588, .018]	.**314** [**− **.021, .586]											
Repetitive motor movement present %	** − .111** [**− **.428, .231]	** − .126** [**− **.441, .216]	** − .120** [**− **.436, .222]	** − .191** [**− **.492, .152]	.**098** [**− **.243, .418]	** − .140** [**− **.452, .202]	** − .070** [**− **.394, .270]	** − .115** [**− **.432, .227]										
Inappropriate utterances^tS^	** − .123** [**− **.438, .219]	** − .111** [**− **.428, .231]	** − .152** [**− **.462, .191]	** − .112** [**− **.430, .229]	** − .247** [**− **.536, .094]	** − .313** [**− **.585, .022]	.**298** [**− **.039, .574]	.**107** [**− **.234, .425]	.**191** [**− **.152, .493]									
Verbal fillers^tS^	− .457** [**− **.686, ** − **.146]	**.332** [**− **.001, .599]	.**064** [**− **.275, .389]	.**039** [**− **.298, .368]	.**236** [**− **.106, .528]	− .377* [**− **.631, ** − **.050]	.385* [.060 .637]	** − .007** [**− **.340, .327]	** − .036** [**− **.365, .301]	** − .129** [**− **.443, .213]								
Repetitive motor movement intensity %	** − .096** [**− **.416, .245]	** − .103** [**− **.422, .239]	** − .209** [**− **.507, .134]	** − .247** [**− **.536, .094]	.**166** [**− **.177, .473]	− .347* [**− **.610, ** − **.016]	** − .093** [**− **.414, .248]	**.051** [**− **.287, .377]	.846*** [.714, .920]	**.299** [**− **.038, .575]	** − .015** [**− **.346, .320]							
Verbal intonation^tL^	** − .022** [**− **.352, .314]	** − .011** [**− **.343, .323]	** − .178** [**− **.483, .165]	** − .140** [**− **.452, .203]	** − .156** [**− **.465, .187]	**.025** [**− **.311, .355]	** − .293** [**− **.571, .044]	** − .252** [**− **.540, .089]	.**212** [**− **.131, .509]	** − .249** [**− **.537, .092]	.**107** [**− **.235, .425]	.**172** [**− **.171, .478]						
Representational gestures intensity %	− .411* [**− **.655, ** − **.091]	** − .181** [**− **.485, .162]	.**000** [**− **.333, .333]	.**053** [**− **.285, .379]	.**191** [**− **.152, .493]	** − .116** [**− **.433, .226]	.**158** [**− **.185, .467]	** − .100** [**− **.419, .241]	.**068** [**− **.272, .392]	** − .062** [**− **.388, .277]	.562*** [.281, .754]	.**024** [**− **.312, .355]	** − .013** [**− **.344, .322]					
Verbal repeats^tS^	** − .333** [**− **.600, .000]	.**057** [**− **.281, .383]	.**191** [**− **.152, .493]	.**210** [**− **.133, .507]	** − .141** [**− **.453, .202]	** − .237** [**− **.529, .104]	**.105** [**− **.237, .423]	.552*** [.268, .748]	.**099** [**− **.243, .418]	.**317** [**− **.018, .588]	.**131** [**− **.212, .445]	.**201** [**− **.141, .501]	.**014** [**− **.321, .345]	.**236** [**− **.105, .528]				
Representational gestures present %	− .473** [**− **.696, ** − **.166]	** − .114** [**− **.431, .227]	.**024** [**− **.312, .355]	.**088** [**− **.253, .409]	.**161** [**− **.182, .469]	** − .098** [**− **.418, .243]	.**182** [**− **.161, .486]	** − .057** [**− **.383, .282]	.**039** [**− **.298, .367]	** − .054** [**− **.381, .284]	.566*** [.287, .756]	.**000** [**− **.333, .334]	.**019** [**− **.317, .350]	.980*** [.961, .990]	.**292** [**− **.046, .570]			
Non- representational gestures present %	** − .264** [**− **.549, .076]	** − .140** [**− **.452, .203]	.**051** [**− **.287, .378]	.**080** [**− **.260, .402]	.**032** [**− **.304, .362]	.**232** [**− **.109, .525]	.**204** [**− **.138, .503]	** − .170** [**− **.477, .173]	** − .045** [**− **.373, .292]	** − .005** [**− **.381, .284]	.**214** [**− **.128, .511]	** − .206** [**− **.505, .137]	** − .242** [**− **.532, .099]	.**205** [**− **.137, .504]	** − .061** [**− **.386, .278]	.**208** [**− **.134, .506]		
Articulation rate	** − .031** [**− **.360, .306]	** − .273** [**− **.556, .066]	.**246** [**− **.095, .536]	.**200** [**− **.143, .500]	** − .071** [**− **.395, .269]	** − .023** [**− **.353, .313]	** − .177** [**− **.481, .166]	.**178** [**− **.165, .483]	.**086** [**− **.255, .407]	.**040** [**− **.297, .368]	** − .127** [**− **.442, .215]	.**156** [**− **.187, .465]	.412* [.091, .566]	** − .062** [**− **.387, .277]	.**274** [**− **.065, .557]	** − .063** [**− **.388, .276]	** − .068** [**− **.392, .272]	
Non- representational gestures intensity %	** − .236** [**− **.528, .105]	** − .127** [**− **.442, .215]	** − .027** [**− **.357, .309]	.**057** [**− **.281, .383]	.**133** [**− **.210, .446]	.**111** [**− **.231, .428]	.**175** [**− **.168, .480]	** − .178** [**− **.483, .165]	** − .011** [**− **.343, .324]	** − .035** [**− **.364, .302]	**.237** [**− **.104, .529]	** − .105** [**− **.423, .236]	** − .160** [**− **.468, .183]	**.296** [**− **.041, .573]	** − .053** [**− **.380, .285]	**.296** [**− **.041, .573]	.934*** [.872, .966]	** − .052** [**− **.379, .286]

The correlation coefficients are highlighted in bold to enhance readability

* = *p* < .05, ** = *p* < .01, *** = *p* < .001

### Exploratory Analysis of Gender Differences

The two-way MANOVA revealed a main effect of autism, *F*(19, 47) = 2.32, *p* = .010, η_*p*_^2^ = 0.484, but no statistically significant main effect of gender, *F*(38, 96) = 1.52, *p* = .054, η_*p*_^2^ = 0.380, or interaction, *F*(19, 47) = 1.47, *p* = .141, η_*p*_^2^ = 0.373. Nevertheless, the effect size indices for both the gender main effect and the interaction term demonstrated potentially strong effects. The follow-up one-way ANOVA descriptive and inferential statistics are reported in Supplementary Materials S10. Given the resulting very small sample sizes for each sub-group—males (autism = 23, no autism = 20), and females (autism = 20, no autism = 20)—we only discuss patterns of findings where the effect size for diagnosis differed in a meaningful way (rather than whether statistical significance changed) from that detected with the full samples.

Autism diagnosis had a medium sized effect on the presentation of seven behaviors for male participants, but only three for females. In addition to increased verbal hesitations and literal interpretation of language, autistic males produced flatter facial expression presence and intensity, fewer shifts in gaze, and less repetitive speech than non-autistic males. The corresponding effects of diagnosis on each of those behaviors for females were only negligible or weak. For females, medium sized effects of diagnosis demonstrated that autistic females restarted their verbalizations and averted their gaze more than non-autistic females, but the corresponding effects for males were weak and negligible. Additionally, medium effect sizes demonstrated greater intonation for male and female autistic adults than non-autistic adults.

Within the autistic sample, only a small number of behaviors differed meaningfully based on gender. Three medium-sized effects demonstrated autistic males produced some behaviors consistent with the diagnosis more than autistic females: namely, flatter facial expression presence and intensity, and more literal interpretation. Another two medium sized effects demonstrated that autistic females displayed some behaviors consistent with the diagnosis more than autistic males: more speech restarts and a faster articulation rate. Among non-autistic adults, there were fewer gender differences identified, with three medium-sized effects demonstrating that males used more verbal fillers, restarted their speech more often, and presented fewer non-representational gestures than females.

## Discussion

We detected a strong effect of autism diagnosis on the overall behavioral pattern displayed within the interview, but only minor differences in the individual observable behaviors of autistic and non-autistic adults. Significant behavioral differences were primarily detected in the domain of social-emotional reciprocity, with autistic adults characterized by more literal interpretation of figurative language (i.e., sarcasm, innuendo, colloquialisms) and longer speech hesitations than for non-autistic adults. Additionally, and unexpectedly, autistic adults demonstrated significantly greater variation in intonation than non-autistic adults. While type I error when interpreting these significant results was possible, Bayes factors provided moderate support for the rejection of the null hypothesis. With larger sample sizes, it is possible that other significant behavior differences—for example, involving behaviors for which the Bayes factors indicated only anecdotal support for the null hypothesis (i.e., flatter facial expression, gaze aversion, increased representational gestures, less repetitive speech)—would emerge that would be mostly consistent with expectations based on diagnostic criteria. However, our data suggest that the effect sizes would likely be extremely weak. Results demonstrated no meaningful effect of diagnosis on memory report, with autistic adults recalling similar levels of accurate details (with certainty) as non-autistic adults, contrary to previous research demonstrating poorer episodic memory recall amongst autistic adults (e.g., Maras et al., 2020).

Further, consistent with previous research suggesting possible differences in the social behavior of male and female autistic individuals (e.g., Tsirgiotis et al., 2024), autistic males displayed more extended verbal hesitations, literal interpretation of language, flatter facial expression and fewer shifts in gaze (consistent with diagnosis), but less repetitive speech and greater intonation (inconsistent with diagnosis) than non-autistic males. For females, fewer behaviors differentiated autistic from non-autistic adults, including increased verbal restarts and gaze aversion (consistent with diagnosis) and greater intonation (inconsistent with diagnosis). Although the examination of gender differences suggests that there may be more noticeable differences in the outward social behavior of autistic males than females, future research will require much larger sample sizes to determine whether these trends are reliable.

Moreover, as well as displaying the inter-individual variability on most behavior measures that is typically found for measures of any psychological construct, the inter-correlation matrix for all behaviors highlighted the within-individual variability among the autistic group. This pattern was highlighted by the absence of significant correlations between behaviors often considered to characterize individuals with an autism diagnosis (e.g., literal interpretation, the presence and intensity of facial expressions, eye gaze behaviors and repetitive movements). Further, although greater variability within the B diagnostic criteria might be expected given not all B criteria need to be met for a diagnosis, the absence of correlations between behaviors in the A domain is surprising given all three criteria must be met for a diagnosis of autism, perhaps reflecting the heterogeneity of the condition even within criteria. Overall, these findings are consistent with the perspective that within a social context many of the behaviors that are expected to distinguish autistic from non-autistic individuals may be less salient as individuals age (e.g., Helt et al., 2008).

### Potential Consequences of Behavioral Differences

The three behavioral characteristics that distinguished the interview performance of autistic adults from that of their non-autistic peers are all potential biasing influences on evaluations of autistic individuals. Difficulties interpreting figurative language may impede individuals’ ability to interact adaptively during social communication. For example, an individual who, during a police interview, literally interpreted a remark such as “do you think I was born yesterday?” and responded “of course not, I would assume you were born more than 30 years ago”, would likely frustrate or antagonize the interviewer (cf. Brewer & Young, 2015, p. 148).

Similarly, lengthier speech hesitations demonstrated by autistic adults may be perceived negatively during some high-stakes interactions. For example, within a criminal justice context, a large body of research demonstrates that confident and non-hesitant recall of interviewees (e.g., suspects, witnesses, defendants; Burgoon et al., 1990; Chalmers et al., 2022; Vrij et al., 2019) shapes observers’ (e.g., law enforcement, judges, jurors) impressions of credibility, deception, and guilt.

The third significant behavioral difference detected between groups—significantly greater variation in intonation of the autistic adults—was inconsistent with expectations based on diagnostic criteria and was also at odds with Lim et al.’s (2022a) finding of minimal systematic group differences in intonation, and Norris et al.’s (2024) finding that observers judged autistic adults’ speech as characterized by monotone intonation. However, it is unclear whether the intonation differences we detected reflected excessive variations in intonation by autistic individuals, particularly flat intonation on the part of non-autistic individuals, or perhaps the greater sensitivity of the software compared to the human ear. Further research using different coding schemes is required to understand observers’ perceptions of the appropriateness of such behaviors in particular contexts.

It is tempting to conclude that the very weak group differences detected on many other behaviors render it unlikely that those behaviors would differentially influence observers’ or interaction partners’ evaluations of autistic and non-autistic individuals. But, as suggested by the sizable group behavior difference highlighted by the multivariate analysis, we do not rule out the possibility that a combination of relatively minor differences across a range of behaviors, including behaviors not captured by our coding system, might have a meaningful cumulative effect. In this context, we are mindful of Lim et al.’s (2022a) finding that adverse judgments of autistic interviewees’ credibility in a forensic interview were predicted by clinicians’ overall impressions even when autistic and non-autistic individuals could not be distinguished based on the prevalence of a number of behaviors thought to reflect an autism diagnosis.

### Limitations

Several aspects of our research paradigm potentially constrain the generality of our conclusions. First, we focused on coding an array of individual behaviors. Yet, the integration of various nonverbal behaviors and speech may be an important determinant of how observers perceive and respond to those with whom they are interacting. Examining this issue will be a potentially valuable focus for future research. Second, other behaviors commonly associated with a diagnosis of autism that could not be examined within our paradigm may shape perceptions of autistic individuals during social interactions. For example, characteristics such as adhering to routine, talking at length about some restricted interest, and displaying sensory sensitivities may be afforded much greater opportunity to manifest during social interactions in many contexts than they were within our online interaction paradigm. To the extent that such behaviors violate expectations of behavior associated with the context in which the interaction is occurring, negative perceptions of the autistic individuals may be more likely (cf. Burgoon, 2015).

Third, the online interview context may moderate any pressures that interviewees might feel in a face-to-face interpersonal interaction, potentially affecting behaviors displayed. For example, research demonstrates autistic individuals report communicative benefits associated with the online context, including a sense of calm during interactions and improved clarity and control over communication (Hassrick et al., 2021). Additionally, whether any participant’s suspicion about the legitimacy of other ‘participants’ in the anonymous online chatroom context might have influenced their interview behavior is unknown. Further, being instructed that the interview would be conducted in a police interview style may have led some participants to modify their demeanor for this context, potentially camouflaging certain behaviors (Cook et al., 2021). Of course, this may accurately reflect camouflaging behavior that might be adapted during a real-world police interaction. Nevertheless, interacting within different in-person and realistic contexts will require individuals to recognize relevant social cues and respond adaptively. Our online paradigm obviously only provided a limited interaction context and, consequently, further research will be needed to establish the generality of our findings.

Fourth, it is possible that demands placed on our participants may have contributed to a degree of self-selection bias, with autistic (and non-autistic) individuals who believed they would be relatively at ease in an interview situation—and in turn may have behaved differently—perhaps more likely to volunteer to participate. Finally, our samples predominantly identified as Caucasian and did not include intellectually disabled individuals, thereby limiting the capacity to generalize conclusions more widely.

## Conclusion

Few significant differences between autistic and non-autistic interviewees were observed in a range of nonverbal and verbal behaviors commonly associated with an autism diagnosis. Although much larger sample sizes may have revealed additional significant differences, the effect size indices suggest that the differences would be relatively weak. Moreover, not only was there considerable inter-individual variability within each group but there was also marked within-individual variability across behaviors, especially for several behaviors commonly associated with the diagnosis. Nevertheless, the overall combination of behavior patterns clearly distinguished the two groups, perhaps increasing the likelihood that observers will perceive autistic adults as behaving differently in potentially high-stakes interviews. To the extent that such differences violate observers’ expectations of appropriate behavior within specific contexts, autistic adults who exhibit those behavioral differences may be the subject of undeserved negative evaluations. Thus, it is important that further research probes whether such behavioral differences are accentuated in face-to-face interactions.

## Supplementary Information

Below is the link to the electronic supplementary material.

Supplementary file1 (DOCX 120 KB)

## Data Availability

The data from the study are available at: https://osf.io/h5uyj/?view_only=95115778d7cd42188ce8accc602d59ad
