# Supplementary material for: The Behavioral Presentation of Autistic Adults in a Forensic Interview
Source: J Autism Dev Disord. 2025 Mar 29;56(9):3503–22. doi: 10.1007/s10803-025-06805-z (PMC13427971; doi:10.1007/s10803-025-06805-z)
Supplement: Supplementary file 1 — Supplementary file1 (DOCX 120 KB) [file 10803_2025_6805_MOESM1_ESM.docx]

**Supplementary Materials S1: Autism Diagnosis Questions for Prolific Participants**

Prolific participants who identified as autistic (*n* = 20) answered the following questions:

1. Have you received a formal diagnosis of autism spectrum disorder from a trained professional?
   1. Yes (*n* = 20)
   2. No (*n* = 0)
2. Please indicate the type of professional(s) who provided the diagnosis:
   1. Psychologist (*n* = 7)
   2. Speech Pathologist (*n* = 1)
   3. Pediatrician (*n* = 2)
   4. Psychiatrist (*n* = 4)
   5. Registered multidisciplinary team: (*n* = 6)
3. What was their name?
   1. Answers censored
4. How old were you when you received the diagnosis?
   1. 0-10 (*n* = 5)
   2. 11-20 (*n* = 5)
   3. 21-30 (*n* = 6)
   4. 31-40 (*n* = 1)
   5. 41-50 (*n* = 2)
   6. 51+ (*n* = 1)
5. Can you describe what happened during the assessment (for example, how long was the session and what did you do?)
   1. Interview/questionnaire (*n* = 18)
   2. Games/activities/tasks/tests (*n* = 14)
   3. Family member interviewed/questioned (*n* = 6)
6. Why did you seek the appointment/why did you see that professional?
   1. Family history (*n* = 1)
   2. Family member/friend suggestion (*n* = 3)
   3. GP referral (*n* = 6)
   4. School referral (*n* = 3)
   5. Behavior (*n* = 10)
   6. Access to services (*n* = 3)
7. Has the diagnosis changed anything? Are there any services that you use now?
   1. Sought services (*n* = 12)
   2. Did not seek services or was unsure (*n* = 8)

**Supplementary Materials S2: Participant Information Received During Study**

- Please consider your level of knowledge about computer/software hacking, and select the option that represents how you feel about the following statement:

| I am knowledgeable about hacking | | | | | |
| --- | --- | --- | --- | --- | --- |
| Strongly disagree | Moderately disagree | Mildly disagree | Mildly agree | Moderately agree | Strongly agree |

- Please read through the information on the following page about hacking. Once you have read through the information, click onto the 'next' page where you will be asked to answer four questions regarding that hacking information.

**Information on Hacking**

**What is hacking?**

- Hacking refers to unauthorized access of a system or network, often to exploit a system’s data or manipulate its normal behavior.
- Now a common part of our vocabulary, we read about hacking daily as data spills and breaches make headlines, and major organizations warn their customers to check their bank statements carefully.
- But while it’s often a catch-all term applied to anything that compromises or negatively affects our computers, ‘hacking’ represents a particular kind of threat to your network and accounts.

**How it works**

- Like breaking into someone’s home, thieves have to look for a way in. Using software code, either developed themselves or available in a ready-to-use kit online, hackers look at ways to gain access to a network. Often finding out a password is the first step in cracking a network’s security.
- Once in, a hacker can modify how a network works, steal data, obtain passwords, get credit card information, watch what you are doing or install software to further the attack.
- While hacking is often highly targeted, some hacking tools, such as ransomware or phishing malware, can spread on their own through links and attachments. Malware can compromise your system or accounts without someone specifically targeting you.

**How to protect yourself from hacking**

- Install anti-virus software on all devices and set it to automatically apply updates and conduct regular scans.
- Always install updates for applications and operating systems when they are available. The longer you delay, the longer you are vulnerable to hackers or malware.
- Use unique, strong passwords for each account (don’t duplicate across accounts) and always use two-factor authentication where possible.
- Always backup your data so if your system is compromised, you won’t necessarily lose everything. Make sure the backup hard drive is not left connected to your system after you’ve finished.
- Always practice safe online browsing behavior and be on the lookout for suspicious links or email attachments.

**What to do if you believe you are a victim of hacking**

- Lodge a report with the XXXX.
- Run a virus scan to identify and remove any malware.
- Change all your passwords and accounts and notify your financial institution/s.
- Notify your social network to be on alert for any strange links or email attachments.

**Questionnaire on the Hacking Information**

- **Based on the information you just read**, please select the **best response** to each of the following questions. If you respond **incorrectly**to any of the following questions, you will be directed back to **read through the hacking information again and to repeat the questions** until you answer each question correctly.

1. Hacking is (select one):
   1. The gaining of unauthorized access to a system or network
   2. Breaking something in half without considering the consequences
   3. Going to lots of different websites as quickly as possible
2. Like breaking into someone’s house, someone can break in to hack a system or a network by (select one):
   1. Using software code, or finding a password
   2. Looking for a key that fits the lock on the door
   3. Calling a friend
3. Some reasons why someone might want to hack someone else are (select one):
   1. To modify how a network works, steal data, obtain passwords, get credit card information, watch what you are doing or install software to further the attack
   2. To play a game on the computer where they get to select a character and complete different levels in a task, meet different people in the game, and advance their gaming skills
   3. To watch their favorite TV show on DVD and share with others which episode they thought was the best one
4. You can protect yourself from hacking by (select all the options that apply):
   1. Installing anti-virus software on all devices and setting it to automatically update and scan the system
   2. Always installing updates for applications and operating systems when they are available
   3. Using strong passwords that are unique across accounts
   4. Backing up your data so you don’t lose everything if your system is hacked
   5. Being cautious when browsing or receiving emails and to not click on or open anything that seems suspicious
   6. Making sure you lock all the doors on your house
   7. Visiting a lot of different websites as quickly as possible
   8. Making sure you have enough battery power when you logon to your computer

If **failed** any of the above:

- You answered at least one of the questions incorrectly, please click the 'next' button at the bottom of this page to read through the information sheet again and then repeat the questions.

If **passed** all of the above:

- You have answered each of the questions correctly. You will now be involved in a short chatroom conversation with other participants who have read through the same information. One of the participants assigned to your chatroom will be randomly selected as the chatroom moderator and given further instructions.

**Information for Chatroom**

- Please enter a username you wish to use to identify yourself in the chatroom. You can use any combination of letters or numbers, but please do not include your first or last name so that you may remain anonymous.
- Please indicate your preferred pronouns:
  1. He/him
  2. She/her
  3. They/them
- Please enter the password hckt19719 below:
- Please click onto the next page to enter the chatroom.

**Supplementary Materials S3: Chatroom Information and Script**

Other participants will join you in this chatroom once they reach the same stage of the session. We do not expect that there will be more than two minutes difference in the session timing between participants, so they should join you shortly.

We request that you remain on this screen until all participants have entered the chatroom. If you have been selected as the moderator, further information will appear below this message regarding chatroom instructions. If you have not been selected as the moderator, please wait for the moderator to begin the chatroom conversation.

Chatroom Users:

**Username: Maze**

Pronouns: He/ Him

**Username: yellow.key**

Pronouns: She/ Her

**Username: S001**

Pronouns: He/ Him

Chatroom Script:

**0:00** (Maze): Hi everyone, I’m going to be the moderator for this chat. So, we are each going to share a bit on what we know about hacking that might be of interest to others. I’ll start us off and then I’ll ask you guys some questions about what you know, and if you don’t feel comfortable responding you don’t have to. If you could wait until I ask you a question before you speak on here that would be great. That’ll keep us from all talking over each other, so it doesn’t get confusing.

(Maze): So, as it happens, I already know a fair bit about hacking because of my previous work. I’ve always been interested in cybersecurity and was hired part time as a penetration tester a year or so ago.

(Maze): Pretty much that job involved me being paid by different companies to hack their network to find flaws in their security so it could be improved. I was there for a bit but have moved onto other security work now while I’m studying

+0.25 **1:45** (Maze): Do any of you have experience with hacking or have anything interesting to share? S001 we’ll start with you?

+0.20 **2:05** (S001): hello. I don’t know much about hacking. I do know a bit about software development. I can code/program etc.

+0.20 **2:25** (S001): penetration testing would have been interesting though.

(S001): was it hard to hack those companies?

+1.20 **3:45** (Maze): Not really, which is why they definitely needed me haha.

(Maze): The job was mostly just a lot of routine checks of servers. But there was some fun stuff involved where I’d get to go undercover and physically enter the company (dressed up as a delivery guy or something like that) to try and access and hack into the server. That was definitely the coolest part of the job, but I didn’t get to do much of that sort of stuff

+0.15 **4:00** (S001): that’s cool.

+0.05 **4:05** (Maze): What about you yellow.key, anything interesting about hacking to share?

+0.25 **4:30** (yellow.key): Well I’ve been hacked before! Wasn’t very interesting for me since I had a bunch of data stolen!!

+0.15 **4:45** (Maze): What do you mean? That sounds like a great time!

+0.20 **5:05** (yellow.key): Hahaha yeah luckily I was able to recover the data!

+0.25 **5:30** (Maze): That’s good you got it all back. Did you know how to do that yourself or did you have to pay someone?

+0.45 **6:15** (yellow.key): I did it myself cos I’ve got a bit of experience in systems management. But after that I definitely went and upgraded my software in case I got hacked again.

+0.25 **6:40** (Maze): Yeah it’s worth it to pay a bit more for that

+0.15 **6:55** (yellow.key): I’d like to see someone try and hack me now!!

+0.10 **7:05** (Maze): Is that a challenge!?

+0.10 **7:15** (yellow.key): Not for you hahaha.

+0.10 **7:25** (Maze): Sorry looks like we’ll have to wrap up soon. (Participant’s username) have you ever been hacked?

[Participant responds]

(Maze): Oh that sucks *OR* that’s good!

(Maze): Ok thanks I think we might leave it here actually. Thanks everyone for your responses! Bye!

**Analysis of Participant Perceptions of Chatroom Users**

SM Table 1 demonstrates participant responses to the debriefing question following the interview (see Supplementary Materials S4): “During the time when you were participating in the chatroom conversation, did you believe the other individuals were real participants?”. A chi-square analysis demonstrated perceptions of participant legitimacy did not differ between autistic and non-autistic participants, χ^2^ (2, 84) = 1.86, *p* = .395, φ = .149 [-.068, .352].

SM Table 1

*Percentage (and number) of participants who provided each response to the question “During the time when you were participating in the chatroom conversation, did you believe the other individuals were real participants”*

| Diagnosis | Yes | No | Unsure |
| --- | --- | --- | --- |
| Autistic (*n* = 43) | 20.9% (9) | 51.2% (22) | 27.9% (12) |
| Non-autistic (*n* = 41) | 29.3% (12) | 36.6% (15) | 34.1% (14) |
| Total (*n* = 84) | 25.0% (21) | 44.0% (37) | 31.0% (26) |

**Supplementary Materials S4: Interview Information and Schedule**

Thank you for your participation in the chatroom. Each participant will now be interviewed individually by a member of the research team.

Police will often investigate instances of suspicious activity online. Given that we are researching discussions of cybercrime, the following interview will be conducted in the manner of an investigation into suspicious online activity, with the interviewer taking on the role of a police investigator and asking questions about the chatroom conversation and your knowledge regarding cybercrime.

When the arrow appears at the bottom of this page please click onto the next page for information to access the Zoom interview.

**Interview Schedule**

Ok, I’m going to ask you some questions now about your general knowledge on hacking and the chatroom conversation. I ask that you answer honestly and in as much detail as you can remember.

For the recording, todays date is >date<. I’m going to begin with some background questions.

1. What is your full name?
2. How old are you?
3. What nationality are you?

I’m going to ask you some questions about your knowledge regarding cybercrime.

1. Can you tell me what hacking is and how hacking can occur?
2. Ok, can you walk me through some reasons someone would want to hack someone else’s information?
3. How could someone go about protecting themselves from hacking?

I’ve got a log from the chatroom here and I’m going to ask you some questions about that.

1. Can you confirm that you were in a chatroom that you accessed online on the >date<?
2. Did you use a password to access the chatroom?
3. Did you use the username >name<?
4. In that particular chatroom conversation did you talk with another member who had the username “Maze”?
5. Were there any other members in that chatroom, and if so, how many?
6. What was the topic that was being discussed in the chatroom?
7. Please tell me as much as you can about Maze and what he spoke about in that chatroom conversation?
8. I can see here the names of the other members were ‘S001’ and ‘yellow.key’, what did they discuss in the chatroom?
9. From the log we can see that in response to the question about whether they had anything interesting to share, yellow key said that they had been hacked and that quote “it wasn’t very interesting for me since I had a bunch of data stolen”. Maze replied to this with “what do you mean, that sounds like a great time!”. What do you think maze meant by that response?
10. In the log we can see that yellow key said that since being hacked they updated their software and would quote “like to see someone try and hack me now!” What do you think they meant by that?
11. Did anyone mention the username “LLTB” in the chatroom?
12. What questions did Maze ask you in this chatroom?
13. Did you answer the question honestly?
14. Overall, did you trust what the other chatroom users said to be true? And can you explain why or why not?

**Debriefing questions answered following the interview (via Qualtrics survey)**

1. Did anything about this study seem strange or odd to you? If yes please specify:
   1. Yes (with open text box response)
   2. No
2. During the time when you were participating in the chatroom conversation, did you believe the other individuals were real participants?
   1. Yes
   2. No
   3. I was unsure
3. Please provide any additional comments you may have about the study in the text box below:
   1. Open text box response

**Supplementary Materials S5: Behavior Coding Instructions**

**Repetitive Body Movement and Gestures**

Instructions:

Partial interval recording of gestures and repetitive body movements requires focusing on whether the behavior did or did not occur during a specified time period. While expressive gestures fall under the category of paralinguistic affect, it will be easiest to code these behaviors concurrently with repetitive body movement as there may be some overlap.

Expressive gestures are operationalized as deliberate movements of the body made when expressing ideas or as a replacement for speech. Gestures include upper body movement (e.g., hands, arms, head, shoulders) used in conjunction with one’s speech, or used as a replacement for speech which may be representational and relate to what is being said (e.g., shrugging shoulders to express uncertainty, nodding head to indicate “yes”, holding up 2 fingers to indicate the number of other chatroom users, demonstrating a behavior that is being described) or non-representational and are not connected to the verbal statement (e.g., clicking fingers, nodding head without referring to “yes” or “no”, moving arms while referring to the number of chatroom users, nodding to indicate they have finished answering the question). Non-examples of gestures include any facial expression, functional movement (e.g., stretching arms, scratching or moving hair off one’s face), or non-expressive movement (e.g., tapping foot or fidgeting with hands). Gestures may be singular or repetitive.

Repetitive body movement is operationalized as any body movement (not including eye movement) or use of object that (1) was not required to meet the demands of the interview or did not appear to serve a functional purpose, and (2) was displayed more than once successively within the duration of the interview. Examples of repetitive movement include running their hand through their hair multiple times, twiddling their thumbs repeatedly, shaking their hands repeatedly, twirling a pen repeatedly, or touching their face multiple times. Non-examples include gesturing to illustrate a point to the interviewer, swatting a fly, sneezing, shifting once to be more comfortable or eyes darting off to the side more than once.

The interview has been broken down into 10-second intervals. Only code a gesture/repetitive movement if it was present during an interval, otherwise leave that cell blank. Code whether the behavior was a repetitive behavior, a representational gesture or a non-representational gesture, and also rate the intensity of that behavior as a 1 (subtle), 2 (moderate) or 3 (intense). Importantly, representational or non- representational gestures may also be repetitive (repeated more than once successively) during an interval. Where this occurs, place an asterisk (*) next to the intensity number you enter for the gesture within that interval. Please note that nodding only counts as a repetitive gesture if the head is bowed down and up more than twice in a row (e.g., head movement down and up and down and up would only be counted as a single gesture).

When determining the intensity of the behavior, focus on how noticeable the behavior was rather than the level of repetitiveness within that interval. An example of a subtle behavior includes when the individual slowly or quickly twiddles their thumbs while their hands are mostly stationary, scratches their finger while their hands are in their lap, or a gesture that is a small shrug of the shoulders or a small nod. Moderate behavior includes more noticeable behavior such as reaching up to scratch their head more than once, moving their hands around in their lap, largely nodding their head twice, or gestures such as largely nodding the head, or moving the hands around out of the lap. Intense behavior includes very noticeable behavior such as rocking the whole body back and forth, moving the whole body around in the chair more than once in a similar motion, or a gesture that uses the full arms to indicate the size of something or circling of the hands while using the arms as well. If you are unable to decide between two intensity ratings, make the lower (i.e., more socially appropriate) rating.

There may be repetitive movement, a representational gesture and non-representational gesture present during an interval, and you can indicate this by coding all types of behaviors and their intensity. Each interval should have a maximum of only one rating in each column for each behavior category, regardless of how many times the behavior occurred within that 10-second period. If the behavior occurred at different intensities within the same 10-second interval, enter the highest intensity rating for that interval. If the behavior did not occur, leave that cell blank.

Note the interval number of the last interval of the interview and record that number under “G. Total Number of Intervals” (include partial intervals; e.g., if the duration of the interview is 7:07, consider 7:06 – 7:10 as the last interval and record “86” as the total number of intervals).

For intensity, an overall score was calculated by dividing the total intensity score (i.e., the sum of all intensity ratings) by the maximum intensity score (i.e., the total number of intervals multiplied by three). This number was then converted into a percentage by multiplying by 100%.

Summary:

| **Behavior**: Repetitive Body Movements |
| --- |
| **Definition of Behavior:** Repetitive body movement is operationalized as any body movement (not including eye movement) or use of object that (1) was not required to meet the demands of the interview or did not appear to serve a functional purpose, and (2) was displayed more than once successively within the duration of the interview  (2) was displayed more than once within the duration of the interview. |
| ***Examples:*** running their hand through their hair multiple times, twiddling their thumbs repeatedly, shaking their hands repeatedly, twirling a pen repeatedly, or touching their face multiple times |
| ***Non-examples:*** gesturing to illustrate a point to the interviewer, swatting a fly, sneezing, shifting once to be more comfortable or eyes darting off to the side more than once |
| **Behavior:** Gestures (representational and non-representational) |
| **Definition of Behavior:** deliberate movements of the body made when expressing ideas or as a replacement for speech. Gestures include upper body movement (e.g., hands, arms, head, shoulders) used in conjunction with one’s speech, or used as a replacement for speech |
| ***Examples representational:*** Relevant to what is being said (e.g., shrugging shoulders to express uncertainty, nodding head to indicate “yes”, holding up 2 fingers to indicate the number of other chatroom users, demonstrating a behavior that is being described) |
| ***Examples*** ***non-representational:*** Irrelevant and not connected to the verbal statement (e.g., clicking fingers, nodding head without referring to “yes” or “no”, moving arms while referring to the number of chatroom users, nodding to indicate they have finished answering the question)) |
| ***Non-examples:*** any facial expression, functional movement (e.g., stretching arms, scratching or moving hair off one’s face), or non-expressive movement (e.g., tapping foot or fidgeting with hands) |

**Facial Expression**

Instructions:

Partial interval recording of facial affect requires focusing on the level of the behavior during a specified time period. Facial affect is operationalized as any change in the face from the neutral baseline expression to a positive or negative expression. Examples of facial expression include a smiling mouth or a furrowed brow, and non-examples include closing the eyes for a period of time, averting gaze, shaking the head, fluttering the eyelids, opening the mouth or holding the mouth open. While some individuals may frown while they are thinking about an answer, this would still be coded as a facial expression. That is, an expression is recorded even if it is to serve a purpose other than to display an emotion (e.g., scrunching up nose to itch it would be coded as an expression).

The interview has been broken down into 10-second intervals. At each interval, indicate whether a facial expression was present during that 10-second period and rate the intensity of the expression:

1 = Subtle

2 = Moderate

3 = Intense

When determining the intensity of the behavior, focus on how noticeable the behavior was. An example of a subtle facial expression includes when the individual displays a small frown for 1 second, or a very subtle frown for 5 seconds. A moderate facial expression includes more noticeable behavior such as raising one's eyebrows for longer than a second. Intense behavior includes very noticeable behavior such as a wide smile or frowning while also pouting one’s lips, this may last for only 1 second or longer. If you are unable to decide between two intensity ratings, make the lower (i.e., more socially appropriate) rating.

Each interval should have a maximum of only one rating, regardless of how many times the behavior occurred within that 10-second period. If the same behavior occurred at different intensities within the same 10-second interval, enter the highest intensity rating for that interval. If there were two separate expressions that occurred during a 10-second period that were both an intensity of 1, you would still score that interval as a 1 rather than combining those two expressions together to score it as a 2. If the same expression is maintained over two intervals (e.g., a continuous smile) you would count that expression in both intervals with an intensity rating. If the behavior did not occur during an interval, leave that cell blank.

Observe and record facial expressions displayed by the individual in all subsequent 10-second intervals in the same way.

Note the interval number of the last interval of the interview and record that number under “C. Total Number of Intervals” (include partial intervals; e.g., if the duration of the interview is 7:07, consider 7:01 – 7:10 as the last interval and record “43” as the total number of intervals).

For intensity, an overall score was calculated by dividing the total intensity score (i.e., the sum of all intensity ratings) by the maximum intensity score (i.e., the total number of intervals multiplied by three). This number was then converted into a percentage by multiplying by 100%.

Summary:

| **Behavior**: Facial Affect |
| --- |
| Description: any movement of the facial features that is different from the neutral baseline expression |
| Example: frowning, furrowing brow, grimacing, pouting lips, smiling, raising eyebrows |
| Non-example: closing eyes, looking away or up, shaking the head, fluttering eyelids, opening the mouth |

**Gaze**

Instructions:

Duration recording of direct gaze requires focusing on the amount of time the individual spent engaged in the behavior. This behavior is operationalized as the individual maintaining their gaze directly into the camera (where the interviewer's face would be appearing on the individual's screen) with no shifts in gaze. Observe and record the start and end of each instance the individual maintained direct gaze at the interviewer. Any shifts in gaze will signal the end of this behavior. Each video will display a timestamp. Record the timestamp of the start and end of the behavioral occurrence in the form provided on the next page. If the behavior occurs for less than 1 second, record the timestamp of the second that directly follows the behavior (i.e., the occurrence should be rounded up to 1 second).

The percentage of the interview the participant displays shifts in gaze is then calculated as a count of the number of occurrences in which the participant averted gaze, divided by the total interview duration.

Summary:

| **Behavior**: Direct Gaze |
| --- |
| **Definition of Behavior:** The individual gazes at the face of the interviewer, without displaying any shifts of gaze. |

**Verbal Content**

Instructions:

The coding of verbal behavior requires focusing on the content of the individual's verbal responses throughout the interview. In particular, five types of verbal behavior will be examined: literal interpretation of language, socially inappropriate responses, immediate echolalia, idiosyncratic phrases, and tangential speech.

**Literal Interpretation of Language**

Definition: A response that suggests the individual has interpreted a statement in an overly literal manner, to the point that the speaker's intention was not understood.

Scoring: The interviewer used/referred to three figurative statements throughout the course of the interview. Code the individual's response to each statement: 2 (did not understand the speaker's intended figurative meaning), 1 (some difficulty understanding the speaker's intended figurative meaning), or 0 (no difficulty understanding the speaker's intended figurative meaning).

- *"Can you tell me what hacking is”*

0 = Provides explanation of hacking

1 = Responds "yes I can" and then provides explanation for what hacking is and how it can occur

2 = Responds with some version of affirmation they can tell you, but does not describe what hacking is, e.g., "yes I can"

- *"Can you walk me through some reasons someone would want to hack someone else’s information?"*

0 = Provides a reason for hacking

1 = Responds "yes I can" and then provides a reason for hacking"

2 = Reference to walking, reference to not understanding the meaning of the question, or responds with only "yes I can"

- *"Maze replied to this with “what do you mean, that sounds like a great time!”. What do you think maze meant by that response?”*

0 = Reference to sarcasm, joking, making light of the situation (or equivalent) OR reference to it being a great time from the perspective of a hacker

1 = General reference to Maze not meaning it but no further explanation given

2 = Reference to incorrect or irrelevant information (e.g., "he thought it sounded fun"), unsure, not understanding

- *" In the log we can see that yellow key said that since being hacked they updated their software and would quote “like to see someone try and hack me now!” What do you think they meant by that?”*

0 = Reference to yellow.key being confident in her security systems (or equivalent)

1 = Reference to yellow.key not meaning it/joking/sarcasm but no further explanation given

2 = Reference to yellow.key wanting to be hacked, unsure, not understanding

**Socially Inappropriate Response**

Definition: A question or statement that is rude, unsuitable, or improper for the context of the interaction.

Scoring: Count the number of questions or statements made by the participant that were socially inappropriate by recording the timestamp at the start of each sentence (each sentence is counted as one occurrence, regardless of the number of words in the question or statement). Also record each statement verbatim in the text box provided.

Examples:

“Duh, of course I was in a chatroom, you already know that”

“I don’t like this interview”

“I thought Maze sounded like an idiot”

"What kind of question is that?"

Non-examples:

“I was suspicious of Maze”

“I didn't think it was very smart for yellow.key to ask to be hacked”

“What was the question?”

**Repeats and Restarting of Speech (of self)**

Definition: The meaningless repetition of words or phrases immediately after their occurrence. Where a single word is repeated, this is a ‘repeat’, where a phrase is repeated this is a ‘restart’.

Scoring: Count the number of times the individual repeats their own response (entirely or only partly) verbatim and immediately after it said by recording the timestamp at the start of each repeat or restart (each restart is counted as one occurrence, regardless of the number of words in the phrase being repeated). Also record each word repeated, or each statement restarted, verbatim in the text box provided.

Example:

"Hacking does occur, hacking does occur… Hacking can occur by illegally accessing a system" (1 restart “hacking does occur”)

“Uhm, uhm, ahh I think the answer is hacking” (1 repeat for “uhm”)

Non-examples:

“Hacking does occur… Hacking can occur by illegally accessing a system” (the second part of the phrase was not an exact repetition, so this does not count as a restart)

“Uhm, ahh I think the answer is hacking” (did not use the same filler word)

**Echolalia – Immediate**

Definition: As above, includes the meaningless repetition of words or phrases immediately after their occurrence, or within the response to the interviewer’s question where the repeated word/phrase comes from, but only those spoken by the interviewer and not the self. This can include both a single word and a phrase.

Scoring: Count the number of times the individual repeats the interviewer’s speech (entirely or only partly) verbatim, and record the timestamp at the start of the word or phrase (like a restart, each instance of echolalia involving more than one word in a phrase is counted as one occurrence, regardless of the number of words in the phrase being repeated). Also record each word repeated, or each statement restarted, verbatim in the text box provided. Where a single word is repeated, but it is not immediate and the context of the word makes sense to repeat to be able to answer the question, this does not count as echolalia.

Examples:

Interviewer: “How can hacking occur?”

Participant: "Hacking occur, hacking occur… Hacking can occur by illegally accessing a system" (2 instances of immediate echolalia “hacking occur”)

Interviewer: “How can hacking occur?”

Participant: “Uhm, uhm, ahh I think the answer is hacking, hacking occur” (1 instance of immediate echolalia for “hacking occur”)

Non-examples:

Interviewer: “How can hacking occur?”

Participant: “Hacking does occur… Hacking can occur by illegally accessing a system” (the second part of the phrase was not an exact repetition of what the interviewer stated, so this does not count as immediate echolalia)

Interviewer: “How can hacking occur?”

Participant: “Uhm, ahh I think the answer is hacking” (while the interviewer used the word hacking, and so did the participant, this was necessary to answer the question and would not be considered out of context as it was used in the sentence)

Interviewer: “How can hacking occur?”

Participant: “Hacking can occur by illegally accessing a system”

Interviewer: “Ok, can you walk me through some reasons someone would want to hack someone else’s information?”

Participant: “Hacking occur, hacking occur, they might want to steal data.” (the echolalia did not come immediately in the participant’s response after the interviewer verbalized the phrase “hacking occur”, it came after the interviewer verbalized another question that did not include the phrase. It does not count if it comes at a later time in this way)

**Idiosyncratic Phrases**

Definition: Using standard words or phrases in an unusual way such that the intended meaning is not readily apparent to the listener

Scoring: Count the number of idiosyncratic phrases used throughout the interview by recording the timestamp at the start of each sentence (each sentence is counted as one occurrence, regardless of the number of words in the sentence). Also record each sentence verbatim in the text box provided.

Examples:

Referring to a computer as “the box” (e.g., "You should make sure to lock your box so that it doesn't get hacked")

Referring to the chatroom as "the other world" (e.g., "I met Maze in the other world")

Non-examples:

Accurate technical terms that may not be recognizable (e.g., DDOS, malware, phishing)

Slang words that may not be grammatically correct but are widely understood by certain groups of people (e.g., "lit", "sledging", "extra", "basic")

**Tangential Speech**

Definition: Responses that are either (1) completely irrelevant to the topic of conversation, or (2) relevant to the general topic of conversation but not necessary to answer the interviewer’s question

Scoring: Record the timestamp at the start and end of each interval the individual engaged in tangential speech. The behavior ends when either of the following occur: (1) the individual shifts to providing information that is necessary to answer the question asked by the interviewer, or (2) the individual stops talking to allow the interviewer to speak.

Examples:

Discussing what they ate for breakfast that morning

Discussing all the people they know who have antivirus software installed (e.g., "You can protect yourself from hacking by installing a good antivirus software. That's why I always make sure that everyone in my family has antivirus software on their computer, because you really never know what can happen to your devices these days. It's just not safe anymore.")

Discussing how Maze reminds them of someone they know (e.g., "Maze was the moderator of the chat, so he was the one asking most of the questions. He seemed quite outspoken, and he actually reminds me of an old friend of mine - he also used to work in IT and had a good sense of humor.")

Non-examples:

Providing a large amount of information that directly answers the interviewer's question (e.g., Discussing in great detail ways to hack into a system when asked how hacking can occur)

Seeking clarification or assistance (e.g., "Could you please repeat the question?" "Sorry, I can't hear you very well")

**Filler Words**

Definition: Any meaningless sound(s) used during speech to fill silence

Scoring: Count the number of fillers used throughout the interview by recording the timestamp at the start of each filler (each filler is counted as one occurrence). Also record each filler in the text box provided.

Examples:

"uh", "uhm", "erm", "ah"

Non-examples:

"mm-hmm", "nah", "yeah", "yay", "you know", "actually", "like", "basically"

**Automated Acoustic Coding Using Praat Version 6.1 (Boersma & Weenik)**

The Praat program was used to code for verbal fluency (articulation rate and hesitations) and paralanguage cues (intonation). This was achieved by inputting the audio recordings of all interviews into the program (removing the interviewer’s questions), which then provided an analysis of the speech patterns. For each participant, the use of the script by Lennes (2016), described in Lennes et al., (2015), in Praat provided their mean pitch (in Hz), their minimum and maximum pitch, and their mean standard deviation of pitch. The script by de Jong and Wempe (2008), described in de Jong and Wempe (2009), was used in Praat to also calculate the number of syllables uttered by the participant, the phonation time (total time spoken), and the number of unfilled pauses in speech (calculated as no phonation within a 0.5 second period).

These data provided by the program were used to then code for the following behaviors:

**Intonation standard deviation**

This was defined as the level of variation (or intonation) participants used in their speech. For example, a larger average standard deviation would indicate that participant uses greater intonation in their speech, compared to a smaller average standard deviation which would indicate more narrow use of intonation. This was calculated based on the standard deviation of the mean pitch for each participant. That is, the degree to which their pitch varied from their mean pitch throughout their phonation time. This is a valid measure of intonation and has been used in previous research with individuals on the autism spectrum (Diehl et al., 2009).

**Pitch mean**

This was defined as the average level of pitch participants displayed in their speech. This provides a baseline level of pitch to compare the other properties against, and provides some indication for how high or low the participants average level of pitch in their voice is during phonation.

**Articulation rate**

This was defined as the speed at which a participant relayed their speech during the interview. This was calculated based on the number of syllables uttered by the participant, divided by their total phonation time. For example, higher scores indicated participants were speaking more quickly (i.e., they provided a greater number of syllables during the time they spoke) compared to those with lower scores.

**Hesitations**

This was defined as the mean length of silent pauses (more than 0.5 seconds) that participants took when it was their turn to speak during the interview. This was calculated based on the total duration of the participant’s turn during the interview, minus their phonation time during that period, then divided by the total number of pauses (more than 0.5 seconds) that were counted during the participant’s turn during the interview. For example, higher scores on this variable indicated participants were spending longer to begin answering the question, or pausing during the middle of a response, compared to those with lower scores who were not as hesitant in their responses.

**References**

de Jong, N. H., & Wempe, T. (2008). Praat script speech rate.sites.google.com/site/speechrate/.

de Jong, N. H., & Wempe, T. (2009). Praat script to detect syllable nuclei and measure speech rate automatically. *Behavior Research Methods, 41*(2), 385-390. https://doi.org/10.3758/BRM.41.2.385

Diehl, J. J., Watson, D., Bennetto, L., McDonough, J., & Gunlogson, C. (2009). An acoustic analysis of prosody in high-functioning autism. *Applied Psycholinguistics, 30*(3), 385-404. https://doi.org/10.1017/S0142716409090201

Lennes, M. (2016). pitch-distributions: Version 1.3. doi:10.5281/zenodo.45868

Lennes, M., Stevanovic, M., Aalto, D., & Palo, P. (2015). Comparing pitch distributions using Praat and R. *Phonetician*, (111-112), 35-53. http://www.isphs.org/Phonetician/Phonetician_111-112.pdf

**Supplementary Materials S6: Memory Report Coding Instructions**

SM Table 2

*Description of memory report characteristics coded*

| Characteristic | Description |
| --- | --- |
| Detail based on study experience | The number of details relayed to the interviewer based on information provided during the study (either the hacking information, or chatroom conversation). Coded based on whether the detail relayed was accurate or inaccurate. Each additional piece of information relayed by the participant was tallied, for example: “hacking is a process by which someone (breaks in) to (steal someone else’s data). and can (watch what you are doing on your computer)” would attract three accurate points given each of those individual pieces of information were relayed in the hacking information as part of the study. Any repetition of the same detail was not coded. |
| Additional detail not from study | Coded as above, but for detail relayed to the interviewer not based on information provided during the study. For example: “the hacker may (steal your mobile information), your (gaming accounts) and (different game profiles) for games like (Warcraft) or (GTA)” would attract five accurate points given they are correct facts, but not relayed to participants at any point in the study. |
| Uncertainty expressed for detail | Number of times uncertainty in a detail was expressed (e.g., “I don’t know much about that”, “I can’t think of anything else”, “I think the answer is X”) or participant responded that they “don’t know” or are “unsure” about an answer. Cumulatively scored, with each additional expression of uncertainty tallied. For example: “(I think) (hacking is breaking into another person’s system). (I don’t know much else about it).” This would be coded as a 1 for an accurate study detail, and 2 for an uncertain response. Any repetition of the same expression of uncertainty for the same detail was not coded. |

Instructions:

**Accurate or Inaccurate Based on Information Received in Study**

Next to each interview question you will be able to view the potential details that the participant may respond with based on information provided during the study (i.e., in the hacking information and chatroom conversation). Please indicate whether the participant relayed each detail, and whether that detail was accurate or inaccurate based on the information they received, by entering a ‘1’ in the corresponding cell next to that detail in the column labelled ‘Accurate (info)’ or ‘Inaccurate (info)’. If the participant relays the information contained in brackets next to some of the potential accurate responses, this will attract an extra point and you would enter a ‘2’ or more depending on the number of details within brackets that were relayed. If the participant relays an accurate detail about a chatroom user but uses the wrong username, a 1 would be entered in the accurate (info) column and a 1 in the inaccurate (info) column.

**Accurate or Inaccurate Additional Detail**

The participant may also relay information related to hacking or the chatroom conversation that was not provided within the study. If they provide additional detail not listed under the column ‘Accurate responses’, please indicate in the row corresponding to each question the number of extra accurate or inaccurate details the participant provided. You may need to Google some of the more technical additional responses provided by participants to assess whether that information is accurate. Add a point for each additional detail relayed—for example, if the participant says: “the hacker may steal your mobile information, your gaming accounts and different game profiles for games like Warcraft or GTA” this would count as 1 point accurate based on information for “hacker may steal”, and 5 points for accurate additional detail in the row ‘steal data’ for (1) mobile information, (2) gaming accounts, (3) game profiles, (4) Warcraft, (5) GTA.

**Uncertainty in Detail**

The participant may also indicate uncertainty regarding a topic they are questioned about (e.g., “I don’t know much about that”, “I can’t think of anything else”, “I think the answer is X”) or respond that they “don’t know” or are “unsure” about an answer. If they provide this information, enter a 1 under the ‘Uncertain’ column in the corresponding row for that question. If they mention multiple times within a question that they don’t know the answer, add an extra point for each time that occurs. If the participant responds with an accurate answer based on the information provided, but also indicates uncertainty, e.g., “I think hacking is breaking into another person’s system” this would be coded as both a 1 for an accurate response and a 1 for an uncertain response.

**PLEASE NOTE** if any of the information is repeated within the response verbatim or in a manner where it is obvious it is the same piece of detail, the repetition is not coded. For example, if the participant responds with: “hackers steal data by breaking into a system. By breaking into your computer hackers can steal credit card information” you would only code 1 for an accurate response based on information for the row ‘by breaking in’ even though it has been repeated twice within their response. This applies for all other responses whether accurate or inaccurate based on information or additional. However, it does not apply where uncertainty is indicated. If uncertainty is repeated several times in a participants’ response, you must still code each time that occurs.

**Examples**

In response to Question 4, a participant may relay the following: “hacking is a process by which someone breaks in to steal someone else’s data. They can do this by accessing back doors and can watch what you are doing on your computer. I don’t know much about this though, I really don’t know anything about hacking”.

This would attract a ‘1’ under the accurate (info) column next to the following details ‘by breaking in’, ‘steal data’, and ‘watch what you’re doing’. This would also attract a ‘1’ in the question 4 row under the accurate (extra) column given they relayed details about backdoors which are an accurate method to hack an individual but was not provided to participants in the study (as it does not appear on the accurate response column). Additionally, you would enter a ‘2’ under the don’t know column in the question 4 row given that they indicated in two sections of their response that they didn’t know any more answers, or any information about hacking.

If the participant responds that “you can’t hack by stealing someone’s password” you would enter a 1 under the inaccurate (info) column in the row corresponding with the accurate response ‘obtain passwords’.

**Supplementary Materials S7: Transformed Descriptive Statistics**

SM Table 3

*Transformed descriptive statistics for mean (SD) and t-test results comparing autistic and non-autistic adults across memory report characteristics*

|  | Diagnosis | |  |
| --- | --- | --- | --- |
| Memory report | Autistic | Non-autistic | *t*-test results |
| Interview duration (seconds) ^tL^ | 2.75 (0.10), n = 43 | 2.74 (0.11), n = 41 | *t*(82) = 0.70, *p* = .486, *d* = 0.15 [-0.28, 0.58] |
| Total details ^tL^ | 1.80 (0.14), n = 43 | 1.83 (0.11), n = 39 | *t*(80) = 0.98, *p* = .332, *d* = 0.22 [-0.22, 0.65] |
| Total accurate details ^tL^ | 1.77 (1.14), n = 43 | 1.80 (0.11), n = 39 | *t*(80) = 1.06, *p* = .292, *d* = 0.24 [-0.20, 0.67] |
| Accurate study detail | 48.35 (14.42), n = 43 | 53.54 (13.80), n = 39 | *t*(80) = 1.66, *p* = .101, *d* = 0.37 [-0.07, 0.80] |
| Inaccurate study detail ^tS^ | 2.08 (0.64), n = 43 | 2.09 (0.61), n = 39 | *t*(80) = 0.06, *p* = .955, *d* = 0.10 [-0.42, 0.45] |
| Accurate additional detail ^tL^ | 1.06 (0.26), n = 43 | 0.99 (0.26), n = 39 | *t*(80) = 1.15, *p* = .255, *d* = 0.25 [-0.18, 0.69] |
| Uncertainty expressed ^tL^ | 1.01 (0.24), n = 43 | 0.94 (0.27), n = 39 | *t*(80) = 1.31, *p* = .195, *d* = 0.29 [-0.15, 0.72] |

SM Table 4

*Transformed descriptive statistics for mean (standard deviation) and number of participants in analysis, for difference in behavior between groups, categorized by predicted outcomes*

| ***Predicted patterns*** | Diagnosis | |
| --- | --- | --- |
| ***Consistent*** | Autistic | Non-autistic |
| Verbal hesitations (mean seconds)^tL^ | 0.37 (0.06), n = 43 | 0.34 (0.03), n = 39 |
| Literal interpretation (range 0-6)^tS^ | 0.59 (0.68), n = 43 | 0.26 (0.50), n = 39 |
| Facial expression present % | 51.59 ( 20.58), n = 39 | 59.01 (21.35), n = 40 |
| Facial expression intensity % | 26.62 (13.89), n = 39 | 29.93 (14.29), n = 40 |
| Gaze shifts % | 8.95 (3.62), n = 37 | 9.70 (2.87), n = 39 |
| Gaze maintenance % | 60.11 (18.78), n = 37 | 63.75 (16.13), n = 39 |
| Verbal tangents (total seconds)^tL^ | 0.35 (0.61), n = 43 | 0.23 (0.62), n = 39 |
| Verbal speech restarts (count of > 1 word)^tL^ | 0.14 (0.28), n = 43 | 0.10 (0.20), n = 39 |
| Repetitive motor movement present % | 67.25 (25.01), n = 41 | 63.23 (31.57), n = 38 |
| Inappropriate utterances^tS^ | 0.06 (0.30), n = 43 | 0.04 (0.23), n = 39 |
| Verbal fillers^tS^ | 6.03 (1.76), n = 43 | 6.16 (2.11), n = 39 |
| Repetitive motor movement intensity % | 31.16 (14.49), n = 41 | 31.03 (17.71), n = 38 |
| ***Inconsistent*** |  |  |
| Verbal intonation (SD of pitch)^tL^ | 1.79 (0.16), n = 43 | 1.69 (0.16), n = 39 |
| Representational gestures intensity % | 12.62 (6.24), n = 43 | 10.75 (5.78), n = 41 |
| Verbal repeats (count of one word)^tS^ | 0.48 (0.72), n = 43 | 0.71 (0.98), n = 39 |
| Representational gestures present % | 22.12 (9.24), n = 43 | 19.78 (8.97), n = 41 |
| Non-representational gestures present % | 28.09 (15.19), n = 43 | 29.57 (15.41), n = 41 |
| Articulation rate (mean speed) | 4.28 (0.33), n = 43 | 4.26 (0.28), n = 39 |
| Non-representational gestures intensity % | 17.06 (10.55), n = 43 | 17.74 (11.17), n = 41 |

^tL^ transformed to be normally distributed using log transformation

^tS^ transformed to be normally distributed using square root transformation

**Supplementary Materials S8: Comparison of Behavioral Difference Values When Adjusted for DASS-21 Scores and Unadjusted**

SM Table 5

*Unadjusted and Adjusted (for DASS-21 scores) ANOVA results for the effect of diagnosis on behavior*

| ***Predicted patterns*** | ***Unadjusted ANOVA result*** | ***Adjusted ANCOVA results*** | ***Comparison*** |
| --- | --- | --- | --- |
| ***Consistent*** |  |  |  |
| More verbal hesitations | *F*(1, 80) =7.29, *p* =.008, η^2^ =.084 | *F*(1, 79) = 7.17, *p* = .009, η^2^ = .083 | No difference |
| More literal interpretation | *F*(1, 80) =6.02, *p* =.016, η^2^ =.070 | *F*(1, 79) = 7.85, *p* = .006, η^2^ = .090 | Marginally stronger when adjusted |
| Flatter facial expression | *F*(1, 77) =2.48, *p* =.120, η^2^ =.031 | *F*(1, 76) = 0.98, *p* = .324, η^2^ = .013 | Marginally weaker when adjusted |
| Flatter intensity of facial expression | *F*(1, 77) = 1.09, *p* =.300, η^2^ = .014 | *F*(1, 76) = 0.20, *p* = .660, η^2^ = .003 | Marginally weaker when adjusted |
| Fewer gaze shifts | *F*(1, 74) = 1.00, *p* = .320, η^2^ = .013 | *F*(1, 73) = 0.27, *p* = .608, η^2^ = .004 | Marginally weaker when adjusted |
| Less gaze maintenance | *F*(1, 74) = 0.82, *p* = .367, η^2^ = .011 | *F*(1, 73) = 0.57, *p* = .453, η^2^ = .008 | No difference |
| Longer verbal tangents | *F*(1, 80) =0.81, *p* =.372, η^2^ =.010 | *F*(1, 79) = 0.74, *p* = .394, η^2^ = .009 | No difference |
| More verbal restarting of speech | *F*(1, 80) =0.66, *p* =.420, η^2^ =.008 | *F*(1, 79) = 1.50, *p* = .225, η^2^ = .019 | Marginally stronger when adjusted |
| More repetitive motor movement | *F*(1, 77) =0.40, *p* =.530, η^2^ =.005 | *F*(1, 76) = 0.07, *p* = .797, η^2^ = .001 | Marginally weaker when adjusted |
| More inappropriate utterances | *F*(1, 80) =0.21, *p* =.647, η^2^ =.003 | *F*(1, 79) = 0.01, *p* = .930, η^2^ = .000 | Marginally weaker when adjusted |
| Use of fewer verbal fillers | *F*(1, 80) =0.09, *p* =.770, η^2^ = .001 | *F*(1, 79) = 0.04, *p* = .849, η^2^ = .000 | No difference |
| More intense repetitive motor movement | *F*(1, 77) =0.01, *p* =.972, η^2^ =.000 | *F*(1, 76) = 0.09, *p* = .770, η^2^ = .001 | Marginally stronger when adjusted |
| ***Inconsistent*** |  |  |  |
| More varied verbal intonation | *F*(1, 80) =6.45, *p* =.013, η^2^ =.075 | *F*(1, 79) = 3.34, *p* = .071, η^2^ = .041 | Becomes non-significant when adjusted, only marginally weaker |
| More intense representational gestures | *F*(1, 82) =2.02, *p* =.159, η^2^ =.024 | *F*(1, 81) = 0.78, *p* = .379, η^2^ = .010 | Marginally weaker when adjusted |
| Less repetitive speech | *F*(1, 80) =1.43, *p* =.235, η^2^ =.018 | *F*(1, 79) = 1.07, *p* = .304, η^2^ = .013 | Marginally weaker when adjusted |
| More representational gestures | *F*(1, 82) =1.39, *p* =.243, η^2^ =.017 | *F*(1, 81) = 0.46, *p* = .501, η^2^ = .006 | Marginally weaker when adjusted |
| Fewer non-representational gestures | *F*(1, 82) =0.20, *p* =.658, η^2^ =.002 | *F*(1, 81) = 0.47, *p* = .494, η^2^ = .006 | Marginally stronger when adjusted |
| Slower articulation rate | *F*(1, 80) =0.10, *p* =.754, η^2^ = .001 | *F*(1, 79) = 0.00, *p* = .951, η^2^ = .000 | No difference |
| Less intense non-representational gestures | *F*(1, 82) =0.08, *p* =.774, η^2^ =.001 | *F*(1, 81) = 0.39, *p* = .536, η^2^ = .005 | Marginally stronger when adjusted |

**Supplementary Materials S9: Intercorrelation between Behaviors**

SM Table 6

*Pearson r correlation [and 95% confidence intervals for r] between behaviors across all groups (n = 70)*

|  | A | B | C | D | E | F | G | H | I | J | K | L | M | N | O | P | Q | R |
| --- | --- | --- | --- | --- | --- | --- | --- | --- | --- | --- | --- | --- | --- | --- | --- | --- | --- | --- |
| A) Verbal hesitations^tL^ | - | - | - | - |  |  |  |  |  |  |  |  |  |  |  |  |  |  |
| B) Literal interpretation^tS^ | .024  [-.212, .257] | - | - | - |  |  |  |  |  |  |  |  |  |  |  |  |  |  |
| C) Facial expression present % | -.083  [-.312, .155] | .082  [-.156, .311] | - | - |  |  |  |  |  |  |  |  |  |  |  |  |  |  |
| D) Facial expression intensity % | -.086  [-.314, .152] | .098  [-.140, .325] | .953***  [.926, .971] | - |  |  |  |  |  |  |  |  |  |  |  |  |  |  |
| E) Gaze shifts % | -.165  [-.385, .073] | .078  [-.160, .308] | .152  [-.086, .373] | .156  [-.082, .377] |  |  |  |  |  |  |  |  |  |  |  |  |  |  |
| F) Gaze maintenance % | .003  [-.232, .238] | -.306**  [-.504, -.076] | -.065  [-.295, .173] | -.085  [-.313, .153] | -.371**  [-.559, -.149] |  |  |  |  |  |  |  |  |  |  |  |  |  |
| G) Verbal tangents^tL^ | -.144  [-.367, .094] | .312**  [.083, .510] | -.062  [-.293, .176] | -.044  [-.276, .193] | .137  [-.101, .360] | -.264*  [-.470, -.031] |  |  |  |  |  |  |  |  |  |  |  |  |
| H) Verbal speech restarts^tL^ | -.032  [-.265, .205] | .360**  [.137, .549] | .084  [-.154, .312] | .096  [-.142, .324] | .126  [-.112, .351] | -.292*  [-.493, -.061] | .331**  [.104, .525] |  |  |  |  |  |  |  |  |  |  |  |
| I) Repetitive motor movement present % | -.046  [-.278, .191] | -.065  [-.296, .172] | -.158  [-.378, .080] | -.203  [-.418, .033] | -.080  [-.309, .158] | .016  [-.219, .250] | -.065  [-.295, .173] | -.045  [-.277, .192] |  |  |  |  |  |  |  |  |  |  |
| J) Inappropriate utterances^tS^ | -.059  [-.290, .179] | -.032  [-.265, .205] | -.126  [-.351, .112] | -.089  [-.318, .149] | -.216  [-.429, .020] | -.269*  [-.474,  -.036] | .236*  [.001, .446] | .107  [-.131, .334] | .133  [-.105, .357] |  |  |  |  |  |  |  |  |  |
| K) Verbal fillers^tS^ | -.292*  [-.493, -.061] | .269*  [.036, .474] | -.017  [-.251, .219] | -.022  [-.256, .214] | .111  [-.127, .337] | -.376**  [-.561, -.155] | .342**  [.117, .534] | .264*  [.031, .470] | -.168  [-.387, .070] | -.083  [-.312, .155] |  |  |  |  |  |  |  |  |
| L) Repetitive motor movement intensity % | -.070  [-.300, .168] | -.061  [-.292, .177] | -.193  [-.410, .043] | -.212  [-.425, .025] | .025  [-.212, .258] | -.144  [-.367, .094] | -.046  [-.278, .191] | .088  [-.150, .316] | .874***  [.804, .920] | .194  [-.043, .410] | -.038  [-.271, .198] |  |  |  |  |  |  |  |
| M) Verbal intonation^tL^ | .107  [-.131, .333] | .047  [-.190, .279] | -.048  [-.280, .189] | -.011  [-.245, .225] | -.114  [-.340, .124] | -.107  [-.334, .131] | -.120  [-.345, .118] | -.060  [-.291, .177] | .257*  [023, .464] | -.120  [-.345, .119] | .021  [-.215, .255] | .205  [-.031, .420] |  |  |  |  |  |  |
| N) Representational gestures intensity % | -.220  [-.432, .016] | -.093  [-.321, .145] | .120  [-.119, .345] | .140  [-.098, .363] | .222  [-.014, .434] | -.163  [-.383, .075] | -.025  [-.259, .221] | -.046  [-.278, .191] | .084  [-.154, .313] | -.027  [-.261, .209] | .240*  [.006, .450] | .115  [-.124, .341] | .120  [-.118, .346] |  |  |  |  |  |
| O) Verbal repeats^tS^ | -.235*  [-.445, .000] | .205  [-.032, .419] | .009  [-.227, .243] | .036  [-.200, .269] | -.102  [-.329, .136] | -.214  [-.428, .022] | .264*  [.031, .470] | .624***  [.456, .749] | .015  [-.221, .249] | .173  [-.065, .392] | .482***  [.278, .644] | .169  [-.069, .388] | .039  [-.198, .271] | .024  [-.212, .257] |  |  |  |  |
| P) Representational gestures present % | -.256*  [-.463,  -.023] | -.085  [-.313, .153] | .135  [-.103, .359] | .161  [-.077, .381] | .182  [-.055, .400] | -.122  [-.347, .116] | -.053  [-.284, .184] | -.039  [-.272, .197] | .042  [-.195, .274] | -.024  [-.258, .212] | .183  [-.054, .401] | .056  [-.181, .287] | .147  [-.091, .369] | .962***  [.939, .976] | .013  [-.223, .247] |  |  |  |
| Q) Non-representational gestures present % | -.239*  [-.449,  -.004] | -.138  [-.361, .100] | -.035  [-.267, .202] | -.027  [-.260, .210] | .080  [-.158, .309] | .119  [-.120, .344] | .305**  [.076, .504] | -.049  [-.281, .188] | .086  [-.152, .314] | -.021  [-.255, .215] | .173  [-.065, .392] | .026  [-.210, .259] | -.022  [-.256, .214] | .176  [-.061, .395] | .113  [-.125, .339] | .129  [-.109, .353] |  |  |
| R) Articulation rate | -.025  [-.259, .211] | -.195  [-.411, .042] | .137  [-.101, .361] | .145  [-.093, .367] | -.086  [-.314, .152] | .125  [-.113, .350] | -.021  [-.254, .215] | .174  [-.064, .393] | .125  [-.113, .350] | .038  [-.199, .271] | -.138  [-.361, .100] | .158  [-.080, .379] | .288*  [.057, .490] | -.065  [-.296, .173] | .197  [-.040, .413] | -.054  [-.286, .183] | .051  [-.186, .282] |  |
| S) Non- representational gestures intensity % | -.230  [-.441, .006] | -.116  [-.341, .123] | -.074  [-.304, .164] | -.032  [-.265, .204] | .164  [-.074, .384] | .006  [-.229, .241] | .319**  [.090, .515] | .008  [-.227, .242] | .111  [-.127, .337] | -.041  [-.273, .196] | .275*  [.043, .479] | .129  [-.109, .354] | .011  [-.225, .245] | .266*  [.033, .471] | .180  [-.057, .398] | .204  [-.032, .419] | .933***  [.894, .958] | .042  [-.195, .274] |

*Note.* * = *p* < .05, ** = *p* < .01, *** = *p* < .001

SM Table 7

*Pearson r correlation [and 95% confidence intervals for r] between behaviors for non-autistic adults (n = 35)*

|  | A | B | C | D | E | F | G | H | I | J | K | L | M | N | O | P | Q | R |
| --- | --- | --- | --- | --- | --- | --- | --- | --- | --- | --- | --- | --- | --- | --- | --- | --- | --- | --- |
| A) Verbal hesitations^tL^ |  |  |  |  |  |  |  |  |  |  |  |  |  |  |  |  |  |  |
| B) Literal interpretation^tS^ | .111  [-.231, .429] |  |  |  |  |  |  |  |  |  |  |  |  |  |  |  |  |  |
| C) Facial expression present % | .364*  [.035, .622] | -.016  [-.347, .319] |  |  |  |  |  |  |  |  |  |  |  |  |  |  |  |  |
| D) Facial expression intensity % | .406*  [.084, .651] | .023  [-.313, .354] | .969***  [.939, .984] |  |  |  |  |  |  |  |  |  |  |  |  |  |  |  |
| E) Gaze shifts % | -.359*  [-.618, -.029] | -.094  [-.414, .247] | .185  [-.158, .488] | .164  [-.179, .472] |  |  |  |  |  |  |  |  |  |  |  |  |  |  |
| F) Gaze maintenance % | .030  [-.307, .359] | -.380*  [-.633, -.053] | -.374*  [-.629, -.047] | -.377*  [-.631, -.050] | -.354*  [-.615, -.023] |  |  |  |  |  |  |  |  |  |  |  |  |  |
| G) Verbal tangents^tL^ | -.234  [-.526, .108] | .057  [-.281, .383] | -.104  [-.423, .237] | -.044  [-.372, .293] | .104  [-.237, .423] | -.144  [-.456, .199] |  |  |  |  |  |  |  |  |  |  |  |  |
| H) Verbal speech restarts^tL^ | -.137  [-.450, .205] | .470**  [.162, .694] | -.167  [-.474, .176] | -.116  [-.433, .226] | .058  [-.280, .384] | -.203  [-.503, .139] | .333  [.000, .600] |  |  |  |  |  |  |  |  |  |  |  |
| I) Repetitive motor movement present % | -.042  [-.370, .295] | -.082  [-.404, .258] | -.171  [-.477, .172] | -.208  [-.506, .135] | -.246  [-.535, .095] | .216  [-.126, .512] | -.093  [-.414, .248] | .002  [-.332, .335] |  |  |  |  |  |  |  |  |  |  |
| J) Inappropriate utterances^tS^ | - | - | - | - | - | - | - | - | - |  |  |  |  |  |  |  |  |  |
| K) Verbal fillers^tS^ | -.124  [-.439, .218] | .260  [-.080, .546] | -.086  [-.408, .254] | -.076  [-.399, .264] | -.018  [-.349, .317] | -.414*  [-.657,  -.093] | .325  [-.010, .594] | .616***  [.356, .788] | -.251  [-.539, .090] | - |  |  |  |  |  |  |  |  |
| L) Repetitive motor movement intensity % | -.069  [-.393, .270] | -.042  [-.370, .295] | -.179  [-.483, .164] | -.181  [-.485,  .016] | -.124  [-.439, .218] | .077  [-.263, .400] | -.013  [-.345, .321] | .135  [-.208, .448] | .897***  [.805, .947] | - | -.054  [-.380, .285] |  |  |  |  |  |  |  |
| M) Verbal intonation^tL^ | .140  [-.203, .452] | -.090  [-.411, .251] | .155  [-.188, .464] | .157  [-.186, .466] | .022  [-.314, .352] | -.187  [-.489, .156] | -.046  [-.374, .292] | .116  [-.226, .432] | .264  [-.076, .549] | - | -.036  [-.365, .301] | .237  [-.104, .529] |  |  |  |  |  |  |
| N) Representational gestures intensity % | .041  [-.297, .369] | -.067  [-.392, .272] | .293  [-.044, .571] | .261  [-.079, .547] | .319  [-.016, .590] | -.197  [-.497, .146] | -.288  [-.567, .050] | .003  [-.331, .336] | .081  [-.260, .403] | - | -.042  [-.370, .295] | .201  [-.142, .500] | .214  [-.128, .511] |  |  |  |  |  |
| O) Verbal repeats^tS^ | -.118  [-.434, .224] | .449**  .136, .680 | -.147  [-.458, .195] | -.105  [-.423, .237] | -.100  [-.419, .241] | -.244  [-.543, .097] | .429**  [.112, .667] | .814***  [.659, .902] | -.021  [-.352, .314] | - | .695***  [.470, .835] | .153  [-.189, .463] | .106  [-.235, .425] | -.139  [-.451, .204] |  |  |  |  |
| P) Representational gestures present % | .055  [-.283, .382] | -.127  [-.441, .216] | .287  [-.051, .566] | .257  [-.083, .544] | .254  [-.087, .541] | -.128  [-.442, .215] | -.360*  [-.619,  -.030] | -.044  [-.372, .293] | .029  [-.307, .359] | - | -.149  [-.459, .194] | .105  [-.237, .423] | .248  [-.093, .537] | .938***  [.881, .969] | -.203  [-.502, .140] |  |  |  |
| Q) Non- representational gestures present % | -.165  [-.472, .178] | -.074  [-.398, .266] | -.149  [-.459, .194] | -.148  [-.459, .195] | .112  [-.230, .429] | -.062  [-.387, .277] | .457**  [.146, .686] | .155  [-.188, .464] | .211  [-.131, .509] | - | .140  [-.203, .452] | .224  [-.118, .519] | .259  [-.081, .545] | .176  [-.167, .481] | .230  [-.112, .523] | .067  [-.273, .391] |  |  |
| R) Articulation rate | -.053  [-.380, .285] | -.113  [-.430, .229] | .001  [-.332, .334] | .076  [-.264, .399] | -.101  [-.420, .240] | .420*  [.101, .661] | .195  [-.148, .496] | .156  [-.187, .465] | .174  [-.169, .479] | - | -.161  [-.469, .182] | .171  [-.173, .477] | .127  [-.215, .442] | -.084  [-.406, .257] | .144  [-.119, .455] | -.051  [-.378, .287] | .228  [-.114, .521] |  |
| S) Non- representational gestures intensity % | -.197  [-.498, .146] | -.041  [-.369, .297] | -.146  [-.457, .196] | -.130  [-.444, .212] | .179  [-.164, .483] | -.160  [-.469, .183] | .505**  [.207, .718] | .292  [-.046, .570] | .222  [-.120, .517] | - | .304  [-.032, .579] | .318  [-.017, .589] | .232  [-.109, .525] | .269  [-.071, .552] | .331  [-.002, .598] | .137  [-.206, .449] | .932***  [.869, .965] | .176  [-.167, .481] |

*Note.* * = *p* < .05, ** = *p* < .01, *** = *p* < .001

*For row and column J – there was no instance of inappropriate utterance for the non-autistic adults included in this examined sample.*

**Supplementary Materials S10: Interaction between Diagnosis and Gender for Behavior**

SM Table 8

*Transformed mean (standard deviation) and number of participants in each group for individual behaviors between groups, split by gender, categorized in line with the results when not splitting by gender*

|  | Male | | Female | |
| --- | --- | --- | --- | --- |
| Predicted patterns and Behaviors | Autistic | Non-autistic | Autistic | Non-autistic |
| Consistent |  |  |  |  |
| Verbal hesitations (mean seconds)^tL^ | 0.38 (0.06), *n* = 23 | 0.35 (0.04), *n* = 18 | 0.36 (0.06), *n* = 20 | 0.34 (0.03), *n* = 20 |
| Literal interpretation (range 0-6)^tS^ | 0.76 (0.73), *n* = 23 | 0.27 (0.53), *n* = 18 | 0.39 (0.56), *n* = 20 | 0.22 (0.46), *n* = 20 |
| Facial expression present % | 45.59 (16.78), *n* = 21 | 61.24 (23.24), *n* = 19 | 58.59 (22.79), *n* = 18 | 55.44 (19.99), *n* = 20 |
| Facial expression intensity % | 22.29 (9.57), *n* = 21 | 31.46 (15.11), *n* = 19 | 31.66 (16.53), *n* = 18 | 27.31 (12.86), *n* = 20 |
| Gaze shifts % | 8.18 (3.87), *n* = 19 | 9.97 (2.84), *n* = 19 | 9.77 (3.24), *n* = 18 | 9.48 (3.02), *n* = 19 |
| Gaze maintenance % | 63.17 (18.28), *n* = 19 | 61.76 (15.95), *n* = 19 | 56.88 (19.27), *n* = 18 | 67.72 (13.96), *n* = 19 |
| Verbal tangents (total seconds)* ^tL^ | 0.28 (0.60), *n* = 23 | 0.13 (0.38), *n* = 18 | 0.42 (0.63), *n* = 20 | 0.32 (0.79), *n* = 20 |
| Verbal speech restarts (count of > one word)^tL^ | 0.07 (0.15), *n* = 23 | 0.17 (0.26), *n* = 18 | 0.22 (0.38), *n* = 20 | 0.04 (0.12), *n* = 20 |
| Repetitive motor movement present % | 65.08 (27.70), *n* = 21 | 59.89 (29.28), *n* = 19 | 69.53 (22.32), *n* = 20 | 65.59 (34.88), *n* = 18 |
| Inappropriate utterances^tS^ | 0.00 (0.00), *n* = 23 | 0.08 (0.33), *n* = 18 | 0.14 (0.44), *n* = 20 | 0.00 (0.00), *n* = 20 |
| Verbal fillers^tS^ | 6.06 (1.71), *n* = 23 | 6.90 (2.11), *n* = 18 | 5.99 (1.86), *n* = 20 | 5.53 (1.99), *n* = 20 |
| Repetitive motor movement intensity % | 30.70 (16.16), *n* = 21 | 20.62 (18.40), *n* = 19 | 31.64 (12.90), *n* = 20 | 30.60 (17.57), *n* = 18 |
| Inconsistent |  |  |  |  |
| Verbal intonation (SD of pitch)^tL^ | 1.81 (0.19), *n* = 23 | 1.70 (0.21), *n* = 18 | 1.76 (0.13), *n* = 20 | 1.68 (0.11), *n* = 20 |
| Representational gestures intensity % | 11.50 (5.81), *n* = 23 | 10.47 (5.66), *n* = 20 | 13.90 (6.62), *n* = 20 | 10.82 (6.11), *n* = 20 |
| Verbal repeats (count of one word)^tS^ | 0.37 (0.67), *n* = 23 | 0.88 (1.11), *n* = 18 | 0.61 (0.78), *n* = 20 | 0.54 (0.86), *n* = 20 |
| Representational gestures present % | 20.39 (8.77), *n* = 23 | 19.33 (9.12), *n* = 20 | 24.10 (9.59), *n* = 20 | 20.04 (9.23), *n* = 20 |
| Non-representational gestures present % | 28.01 (16.30), *n* = 23 | 25.89 (15.22), *n* = 20 | 28.18 (14.22), *n* = 20 | 33.85 (15.09), *n* = 20 |
| Articulation rate (mean speed) | 4.20 (0.37), *n* = 23 | 4.23 (0.25), *n* = 18 | 4.37 (0.25), *n* = 20 | 4.30 (0.32), *n* = 20 |
| Non-representational gestures intensity % | 17.43 (11.32), *n* = 23 | 16.48 (11.35), *n* = 20 | 16.64 (9.87), *n* = 20 | 19.34 (11.28), *n* = 20 |

^tL^ transformed using log transformation

^tS^ transformed using square root transformation

SM Table 9

*ANOVA outcomes for the effect of autism diagnosis on behaviors for male participants, categorized in line with the results when not splitting by gender with a comparison to the main effect of diagnosis across both genders*

| Behavior Pattern | ANOVA Outcome | Interpretation | Comparison |
| --- | --- | --- | --- |
| Behavior Pattern Consistent with Predicted | | |  |
| More verbal hesitations | *F*(1, 39) = 4.70, *p* =.036 | significant |  |
|  | η^2^ = .108 [.000, .298] | medium effect | consistent |
| More literal interpretation | *F*(1, 39) = 5.66, *p* =.022 | significant |  |
|  | η^2^ = .127 [.001, .320] | medium effect | consistent |
| Flatter facial expression | *F*(1, 38) = 6.04, *p* =.019 | significant |  |
|  | η^2^ = .137 [.003, .334] | medium effect | stronger |
| Flatter facial expression intensity | *F* (1, 38) = 5.37, *p* =.026 | significant |  |
|  | η^2^ = .124 [.000, .319] | medium effect | stronger |
| Fewer gaze shifts | *F* (1, 36) = 2.63, *p* = .114 | non-significant |  |
|  | η^2^ = .068 [.000, .255] | medium effect | stronger |
| Less gaze maintenance | *F* (1, 36) = 0.06, *p* = .801 | non-significant |  |
|  | η^2^ = .002 [.000, .100] | negligible effect | weaker |
| Longer verbal tangents | *F* (1, 39) = 0.84, *p* =.364 | non-significant |  |
|  | η^2^ =.021 [.000, .168] | very weak effect | consistent |
| More verbal speech restarts | *F* (1, 39) = 2.12, *p* =.154 | non-significant |  |
|  | η^2^ =.051 [.000, .223] | weak effect | stronger |
| More repetitive motor movement | *F* (1, 38) = 0.33, *p* =.568 | non-significant |  |
|  | η^2^ =.009 [.000, .137] | negligible effect | consistent |
| More inappropriate utterances | *F* (1, 39) = 1.29, *p* =.264 | non-significant |  |
|  | η^2^ =.032 [.000, .190] | weak effect | stronger, pattern changes direction |
| Fewer verbal fillers | *F* (1, 39) = 1.98, *p* =.167 | non-significant |  |
|  | η^2^ = .048 [.000, .218] | weak effect | stronger |
| More intense repetitive movement | *F* (1, 38) =0.00, *p* =.989 | non-significant |  |
|  | η^2^ =.000 [.000, .000] | negligible effect | consistent |
| Behavior Pattern Inconsistent with Predicted | | |  |
| More varied verbal intonation | *F* (1, 39) = 3.13, *p* =.085 | non-significant |  |
|  | η^2^ =.074 [.000, .256] | medium effect | consistent |
| More intense representational gestures | *F* (1, 41) = 0.35, *p* =.558 | non-significant |  |
|  | η^2^ =.008 [.000, .130] | negligible effect | weaker |
| Less repetitive speech | *F* (1, 39) = 3.29, *p* =.078 | non-significant |  |
|  | η^2^ =.078 [.000, .261] | medium effect | stronger |
| More representational gestures | *F* (1, 41) = 0.15, *p* =.700 | non-significant |  |
|  | η^2^ =.004 [.000, .108] | negligible effect | weaker |
| Fewer non-representational gestures | *F* (1, 41) = 0.19, *p* =.663 | non-significant |  |
|  | η^2^ =.005 [.000, .114] | negligible effect | consistent size, pattern changes direction |
| Slower articulation rate | *F* (1, 39) = 0.08, *p* =.779 | non-significant |  |
|  | η^2^ = .002 [.000, .098] | negligible effect | consistent |
| Less intense non-representational gestures | *F* (1, 41) = 0.08, *p* =.786 | non-significant |  |
|  | η^2^ =.002 [.000, .093] | negligible effect | consistent size, pattern changes direction |

SM Table 10

*ANOVA outcomes for the effect of autism diagnosis on behaviors for female participants, categorized in line with the results when not splitting by gender with a comparison to the main effect of diagnosis across both genders*

| Behavior Pattern | ANOVA Outcome | Interpretation | Comparison |
| --- | --- | --- | --- |
| Behavior Pattern Consistent with Predicted | | |  |
| More verbal hesitations | *F*(1, 38) = 1.98, *p* =.168 | non-significant |  |
|  | η^2^ =.049 [.000, .222] | weak effect | weaker |
| More literal interpretation | *F*(1, 38) = 1.11, *p* =.298 | non-significant |  |
|  | η^2^ =.028 [.000, .186] | weak effect | weaker |
| Flatter facial expression | *F*(1, 36) = 0.22, *p* =.646 | non-significant |  |
|  | η^2^ =.006 [.000, .130] | negligible effect | weaker, pattern changes direction |
| Flatter facial expression intensity | *F*(1, 36) = 0.83, *p* =.369 | non-significant |  |
|  | η^2^ = .023 [.000, .179] | weak effect | consistent size, pattern changes direction |
| Fewer gaze shifts | *F* (1, 35) = 0.08, *p* = .786 | non-significant |  |
|  | η^2^ = .002 [.000, .106] | negligible effect | weaker, pattern changes direction |
| Less gaze maintenance | *F* (1, 35) = 3.87, *p* = .057 | non-significant |  |
|  | η^2^ = .100 [.000, .298] | medium effect | stronger |
| Longer verbal tangents | *F* (1, 38) = 0.21, *p* =.650 | non-significant |  |
|  | η^2^ =.005 [.000, .124] | negligible effect | weaker |
| More verbal speech restarts | *F* (1, 38) = 4.16, *p* =.048 | significant |  |
|  | η^2^ = .099 [.000, .290] | medium effect | stronger |
| More repetitive motor movement | *F* (1, 36) = 0.18, *p* = .677 | non-significant |  |
|  | η^2^ =.005 [.000, .125] | negligible effect | consistent |
| More inappropriate utterances | *F* (1, 38) = 2.00, *p* = .170 | non-significant |  |
|  | η^2^ =.049 [.000, .222] | weak effect | stronger |
| Fewer verbal fillers | *F* (1, 38) = 0.55, *p* =.463 | non-significant |  |
|  | η^2^ = .014 [.000, .154] | very weak effect | stronger, pattern changes direction |
| More intense repetitive movement | *F* (1, 36) = 0.05, *p* =.830 | non-significant |  |
|  | η^2^ =.001 [.000, .093] | negligible effect | consistent |
| Behavior Pattern Inconsistent with Predicted | | |  |
| More varied verbal intonation | *F* (1, 38) = 3.79, *p* =.059 | non-significant |  |
|  | η^2^ =.091 [.000, .280] | medium effect | consistent |
| More intense representational gestures | *F* (1, 38) = 2.34, *p* =.135 | non-significant |  |
|  | η^2^ =.058 [.000, .235] | weak effect | stronger |
| Less repetitive speech | *F* (1, 38) = 0.07, *p* =.792 | non-significant |  |
|  | η^2^ =.002 [.000, .098] | negligible effect | weaker, pattern changes direction |
| More representational gestures | *F* (1, 38) = 1.87, *p* =.180 | non-significant |  |
|  | η^2^ =.047 [.000, .218] | weak effect | stronger |
| Fewer non-representational gestures | *F* (1, 38) = 1.50, *p* =.229 | non-significant |  |
|  | η^2^ =.038 [.000, .203] | weak effect | stronger |
| Slower articulation rate | *F* (1, 38) = 0.64, *p* =.428 | non-significant |  |
|  | η^2^ = .017 [.000, .160] | very weak effect | stronger, pattern changes direction |
| Less intense non-representational gestures | *F* (1, 38) = 0.65, *p* =.426 | non-significant |  |
|  | η^2^ =.017 [.000, .160] | very weak effect | stronger |

SM Table 11

*ANOVA outcomes for the effect of gender on behaviors for autistic adults (male compared to female), categorized in line with the size of the effect*

| Behavior Pattern | ANOVA Outcome | Interpretation |
| --- | --- | --- |
| Flatter facial expression intensity | *F*(1, 37) = 4.87, *p* =.034 | significant |
|  | η^2^ = .116 [.000, .313] | medium effect |
| Flatter facial expression | *F*(1, 37) = 4.19, *p* =.048 | significant |
|  | η^2^ =.102 [.000, .296] | medium effect |
| More literal interpretation | *F*(1, 41) = 3.28, *p* =.078 | non-significant |
|  | η^2^ =.074 [.000, .251] | medium effect |
| Fewer speech restarts | *F* (1, 41) = 2.97, *p* =.092 | non-significant |
|  | η^2^ = .068 [.000, .242] | medium effect |
| Slower articulation rate | *F* (1, 41) = 2.86, *p* =.098 | non-significant |
|  | η^2^ = .066 [.000, .239] | medium effect |
| Fewer inappropriate utterances | *F* (1, 41) = 2.26, *p* = .141 | non-significant |
|  | η^2^ =.052 [.000, .220] | weak effect |
| Fewer gaze shifts | *F* (1, 35) = 1.81, *p* = .187 | non-significant |
|  | η^2^ = .049 [.000, .230] | weak effect |
| Fewer representational gestures | *F* (1, 41) = 1.76, *p* =.192 | non-significant |
|  | η^2^ =.041 [.000, .202] | weak effect |
| Less intense representational gestures | *F* (1, 41) = 1.61, *p* =.212 | non-significant |
|  | η^2^ =.038 [.000, .196] | weak effect |
| More verbal hesitations | *F*(1, 41) = 1.30, *p* =.261 | non-significant |
|  | η^2^ =.031 [.000, .184] | weak effect |
| More gaze maintenance | *F* (1, 35) = 1.04, *p* = .315 | non-significant |
|  | η^2^ = .029 [.000, .194] | weak effect |
| Less repetitive speech | *F* (1, 41) = 1.15, *p* =.290 | non-significant |
|  | η^2^ =.027 [.000, .177] | weak effect |
| More varied verbal intonation | *F* (1, 41) = 1.13, *p* =.293 | non-significant |
|  | η^2^ =.027 [.000, .176] | weak effect |
| Shorter verbal tangents | *F* (1, 41) = 0.59, *p* =.447 | non-significant |
|  | η^2^ =.014 [.000, .147] | very weak effect |
| Less repetitive motor movement | *F* (1, 39) = 0.32, *p* = .575 | non-significant |
|  | η^2^ =.008 [.000, .133] | negligible effect |
| More intense non-representational gestures | *F* (1, 41) = 0.06, *p* =.810 | non-significant |
|  | η^2^ =.001 [.000, .088] | negligible effect |
| Less intense repetitive movement | *F* (1, 39) = 0.04, *p* =.838 | non-significant |
|  | η^2^ =.001 [.000, .085] | negligible effect |
| More verbal fillers | *F* (1, 41) = 0.02, *p* =.888 | non-significant |
|  | η^2^ = .000 [.000, .066] | negligible effect |
| Fewer non-representational gestures | *F* (1, 41) = 0.00, *p* =.972 | non-significant |
|  | η^2^ =.000 [.000, .006] | negligible effect |

SM Table 12

*ANOVA outcomes for the effect of gender on behaviors for non-autistic adults (male compared to female), categorized in line with the size of the effect*

| Behavior Pattern | ANOVA Outcome | Interpretation |
| --- | --- | --- |
| More verbal fillers | *F* (1, 36) = 4.23, *p* = .047 | significant |
|  | η^2^ = .105 [.000, .302] | medium effect |
| More verbal speech restarts | *F* (1, 36) = 3.87, *p* =.057 | non-significant |
|  | η^2^ =.097 [.000, .293] | medium effect |
| Fewer non-representational gestures | *F* (1, 38) = 2.76, *p* =.105 | non-significant |
|  | η^2^ =.068 [.000, .249] | medium effect |
| Less gaze maintenance | *F* (1, 36) = 1.50, *p* = .228 | non-significant |
|  | η^2^ =.040 [.000, .212] | weak effect |
| More inappropriate utterances | *F*(1, 36) = 1.12, *p* =.298 | non-significant |
|  | η^2^ =.030 [.000, .194] | weak effect |
| More repetitive speech | *F* (1, 36) = 1.12, *p* = .297 | non-significant |
|  | η^2^ =.030 [.000, .194] | weak effect |
| Shorter verbal tangents | *F* (1, 36) = 0.88, *p* = .355 | non-significant |
|  | η^2^ = .024 [.000, .181] | weak effect |
| Greater facial expression intensity | *F* (1, 37) = 0.86, *p* =.361 | non-significant |
|  | η^2^ = .023 [.000, .176] | weak effect |
| Greater facial expression | *F*(1, 37) = 0.73, *p* =.398 | non-significant |
|  | η^2^ =.019 [.000, .169] | very weak effect |
| Less intense non-representational gestures | *F* (1, 38) = 0.64, *p* =.430 | non-significant |
|  | η^2^ =.016 [.000, .160] | very weak effect |
| More verbal hesitations | *F*(1, 36) = 0.52, *p* =.475 | non-significant |
|  | η^2^ = .014 [.000, .159] | very weak effect |
| Slower articulation rate | *F* (1, 36) = 0.50, *p* =.486 | non-significant |
|  | η^2^ = .014 [.000, .157] | very weak effect |
| Less repetitive motor movement | *F* (1, 35) = 0.29, *p* =.593 | non-significant |
|  | η^2^ =.008 [.000, .142] | negligible effect |
| More gaze shifts | *F* (1, 36) = 0.26, *p* =.615 | non-significant |
|  | η^2^ = .007 [.000, .135] | negligible effect |
| More literal interpretation | *F*(1, 36) = 0.09, *p* =.768 | non-significant |
|  | η^2^ =.002 [.000, .108] | negligible effect |
| More varied verbal intonation | *F* (1, 36) = 0.08, *p* =.776 | non-significant |
|  | η^2^ =.002 [.000, .106] | negligible effect |
| Fewer representational gestures | *F* (1, 38) = 0.06, *p* =.809 | non-significant |
|  | η^2^ =.002 [.000, .094] | negligible effect |
| Less intense representational gestures | *F* (1, 38) = 0.04, *p* =.848 | non-significant |
|  | η^2^ =.001 [.000, .084] | negligible effect |
| Less intense repetitive movement | *F* (1, 35) = 0.00, *p* =.993 | non-significant |
|  | η^2^ =.000 [.000, .000] | negligible effect |
